# Assessing the impact of probiotics on immunotherapy effectiveness and antibiotic-mediated resistance in cancer: a systematic review and meta-analysis

**DOI:** 10.3389/fimmu.2025.1538969

**Published:** 2025-03-21

**Authors:** Shuya Zhao, Zian Lu, Fangmin Zhao, Shihuan Tang, Lishan Zhang, Cuiling Feng

**Affiliations:** ^1^ Dongzhimen Hospital, Beijing University of Chinese Medicine, Beijing, China; ^2^ Peking University People’s Hospital, Beijing, China; ^3^ Department of First Clinical Medical College, Zhejiang Chinese Medical University, Hangzhou, China; ^4^ State Key Laboratory for Quality Ensurance and Sustainable Use of Dao-di Herbs, Beijing, China

**Keywords:** cancer, immunotherapy, probiotics, antibiotics, meta-analysis, systematic review

## Abstract

**Background:**

Probiotics have been demonstrated to exert a potential clinical enhancing effect in cancer patients receiving immune checkpoint inhibitors (ICIs), while antibiotics exert a detrimental impact. Prior meta-analysis papers have substantial limitations and are devoid of recent published studies. Therefore, this study aimed to perform an updated meta-analysis and, for the first time, assess whether probiotics can restore the damage of antibiotics to immunotherapy.

**Methods:**

A comprehensive literature search was conducted in three English databases and three Chinese databases with a cutoff date of August 11, 2024. The methodological quality of the studies was evaluated using the Newcastle-Ottawa Quality Assessment Scale (NOS) or the Revised Cochrane risk-of-bias tool (RoB 2). Engauge Digitizer v12.1 was employed to extract hazard ratios (HRs) with 95% confidence interval (CI) for survival outcomes when these data were not explicitly provided in the manuscripts. Meta-analysis was conducted using Stata 14 software.

**Results:**

The study sample comprised eight retrospective and four prospective studies, involving a total of 3,142 participants. The findings indicate that probiotics significantly prolong the overall survival (OS) (I^2^ = 31.2%; HR=0.58, 95% CI: 0.46-0.73, p < 0.001) and progression-free survival (PFS) (I^2^ = 65.2%; HR=0.66, 95% CI: 0.54-0.81, p < 0.001) in cancer patients receiving ICIs, enhance the objective response rate (ORR) (I^2^ = 33.5%; OR=1.75, 95% CI: 1.27-2.40, p = 0.001) and disease control rate (DCR) (I^2^ = 50.0%; OR=1.93, 95% CI: 1.11-3.35, p = 0.002). For non-small cell lung cancer (NSCLC) patients exposed to antibiotics, the use of probiotics was associated with superior OS (I^2^ = 0.0%; HR=0.45, 95% CI: 0.34-0.59, p < 0.001) and PFS (I^2^ = 0.0%; HR=0.48, 95% CI: 0.38-0.62, p < 0.001) when compared to non-users. Subgroup differences were observed regarding the cancer type (P=0.006) and ethnic backgrounds (P=0.011) in OS.

**Conclusions:**

The meta-analysis findings suggest that probiotics can effectively extend the survival of cancer treated with ICIs. In NSCLC, probiotics appear to mitigate the negative impact of antibiotics on immunotherapy effectiveness, which has profound clinical significance. Nevertheless, additional large-scale, high-quality randomized controlled trials are necessary to further validate these findings.

**Systematic review registration:**

https://www.crd.york.ac.uk/PROSPERO/display_record.php?RecordID=579047, identifier CRD42024579047.

## 1 Introduction

Immune checkpoint inhibitors (ICIs) dramatically altered the landscape of cancer treatment, and markedly improved the prognosis (1–3), particularly for patients with melanoma and non-small cell lung cancer (NSCLC) (4–6). Nonetheless, therapeutic efficacy of ICIs exhibits considerable variability among cancer patients, with a significant proportion developing primary or secondary resistance during initial treatment. The incidence of primary resistance to ICIs ranges from approximately 10% to 27% (7), while secondary resistance ranges from 52% to 57% (8). Majority of patients develop resistance to ICIs between 3 months to 3 years following the initiation of treatment (9). The mechanisms underlying immunotherapy resistance in NSCLC are multifaceted, encompassing not only intra-tumoral factors such as epigenetic alterations, gene mutations, abnormal signaling pathways, and deficiency in tumor immunogenicity, but also extrinsic determinants like the dysregulation of immune cells (e.g., tumor-associated macrophages (TAMs), B cells, natural killer cell (NK cells), T cells) and the expression of immunosuppressive molecules within the tumor microenvironment (TME) (7, 9, 10). Delaying the onset of immunotherapy resistance and identifying biomarkers for predicting treatment efficacy are particularly crucial.

The gut microbiome, the gut microbiome, known to contain at least 100 times more genes than the human genome, is commonly recognized as the “second genome” of humans (11). Recent studies have demonstrated that the composition of gut microbiome can affect the therapeutic response to ICIs (12), compared to non-responders to ICIs, responders exhibit a more diverse gut microbiota composition and a higher richness of specific microbial communities (12–14). Antibiotics impair the outcomes of ICIs therapy in cancer, likely by disrupting the gut microbiota, resulting in a shorter survival (15–18), a meta-analysis confirmed this conclusion (19). Consequently, regulating the gut microbiota is considered a potential approach to address ICIs resistance (14, 20).

Probiotics, which are live microbial dietary supplements, function to recover the intestinal microbial balance or increases gut microbial diversity (21), the concurrent use of probiotics foster a beneficial immune environment and enhance the efficacy of ICIs (22). Supplementation with Lactobacillus johnsonii has been shown to augment CD8+ T cell-mediated α programmed death-1 (αPD-1) immunotherapy through modulating the stemness program of CD8+ T cells and facilitating the generation of progenitor exhausted CD8+ T cells (23). A meta-analysis of five retrospective studies revealed that probiotics administration was correlated with improved progression-free survival (PFS) (hazard ratio [HR] = 0.51, 95% confidence interval [CI]: 0.42–0.61, p < 0.01) and overall survival (OS) (HR = 0.50, 95% CI: 0.30–0.85, p = 0.01), but did not affect the objective response rate (ORR) (24). Results from a randomized Phase 1 clinical trial indicated a significantly higher ORR in renal cell carcinoma patients treated with CBM588, a bifidogenic live bacterial preparation, than in the control group (14 of 19, 74% versus 2 of 10, 20%; P = 0.01). However, the median OS and PFS were not achieved by the time of data cutoff for both groups (25). The clinical value of co-administering probiotics with ICIs continues to be a subject of debate.

In light of the marked rise in newly published research over the last two years, it is essential to update our understanding of the relationship between probiotics intake and the effectiveness of anti-tumor immunotherapy. Furthermore, cancer patients frequently have a higher likelihood of antibiotic exposure, yet no published meta-analyses exist that examine whether probiotics can ameliorate the detrimental effects of antibiotics on tumor immune responses. Therefore, a new meta-analysis is required, the objectives of this meta-analysis include: 1) updating the relevant data regarding the influence of probiotics supplementation on immunotherapy efficacy; 2) assessing the effect of probiotics on ICIs when patient exposed to antibiotics.

## 2 Methods

This paper was conducted in accordance with the Preferred Reporting Items for Systematic Reviews and Meta-Analyses (PRISMA) guidelines (26). The protocol for this meta-analysis has been registered in PROSPERO (Registration number: CRD42024579047).

### 2.1 Literature search strategy

Two researchers (ZSY and LZA) performed a comprehensive and systematic search across six databases, including PubMed, EMBASE, Cochrane Library, Chinese National Knowledge Infrastructure (CNKI), WanFang, and SinoMed, with a cut-off date of August 11, 2024. Additionally, the citation lists of included studies, previous systematic reviews, Clinicaltrials.gov and Google Scholar were also screened as supplements. The search was restricted to articles published in English and Chinese. The search terms “neoplasms”[mesh], “Immune Checkpoint Inhibitors” [Mesh], “Probiotics” [Mesh], along with their entry terms, were explored within [All Fields] or [Title/Abstract/Keyword], taking the retrieval strategy of PubMed database as an example (**Supplementary Table S1**).

### 2.2 Inclusion and exclusion criteria

Studies that matched all of the following criteria were included: (1) Patients were diagnosed as malignant tumors; (2) Received ICIs (anti-PD-1/anti-programmed cell death ligand 1 (anti-PD-L1)/anti-cytotoxic T lymphocyte-associated antigen-4 (anti-CTLA-4)) either as monotherapy or in combination; (3) According to usage of probiotics before, during, or after the ICIs therapeutic course, patients were categorized into exposed cohort (probiotics use) and non-exposed cohort (no probiotics use); (4) The primary endpoints include OS or PFS; (5) A HR with 95% CI for OS and/or PFS can be extracted or derive from the study data.

Studies that matched any of the following criteria were excluded: (1) Inconsistencies exist in the type of disease research or intervention; (2) Animal studies; (3) Letters, comments, case reports, guidelines, editorials, conference abstracts and reviews; (4) Study outcomes are insufficient or cannot be extracted. Article with the most comprehensive data and rigorous methods is chosen when studies reported overlapping patient populations.

Screening was conducted by two independent reviewers (ZSY and LZA), and any discrepancies after their discussion being adjudicated by a third reviewer (FCL) to reach consensus.

### 2.3 Data extraction

Two reviewers independently extracted the basic data and then cross-verified them separately, resolving discrepancies through discussion. The data extracted included the first author’s name, year of publication, study region and period, study type, study demographics, tumor type, types of ICIs treatment and probiotics, pooled effect size and 95% CI. Newer and more detailed data were used when the same outcome data were provided in both studies. Treatment response was evaluated using the Response Evaluation Criteria in Solid Tumors (RECIST) version 1.1. Complete response (CR), partial response (PR), or stable disease (SD) lasting longer than 6 months was considered disease control. When both univariate and multivariate analyses of HR for OS or PFS were available, the latter was preferred. Engauge Digitizer v12.1 was used to extract survival data from Kaplan-Meier curves and analyzed using the excel program file proposed by Tierney et al. (27). when HRs were not directly provided in the manuscript.

### 2.4 Literature quality evaluation

The Newcastle-Ottawa Quality Assessment Scale (NOS) was adopted to evaluate the quality of the cohort and case-control studies (https://www.ohri.ca/programs/clinical_epidemiology/oxford.asp). Studies with a score ≥7 were deemed to high quality, while those < 6 were classified as low quality. The Revised Cochrane risk-of-bias tool for randomized trials (RoB 2.0) was utilized to assess the methodological quality of randomized controlled trials (RCT) (28). Two reviewers independently assessed the risk of bias, any discrepancies were resolved by reaching a consensus with the assistance of a third reviewer.

### 2.5 Statistical analysis

The primary endpoints of this meta-analysis were OS and/or PFS, while the secondary endpoints were ORR and/or DCR. Statistical analyses were conducted with Stata 14. The pooled HRs/odds ratios (ORs) and their corresponding 95% CI were calculated to assess the impact of probiotics administration on the efficacy of ICIs. Subgroup analyses will be done based on tumor type, survival data will be analyzed separately for patients with and without antibiotic use. The chi-squared (I^2^) test was utilized to evaluate the level of statistical heterogeneity among the included studies. Heterogeneity was classified as high (I^2^ ≥ 50%), moderate (20% ≤ I^2^ < 50%), or low (I^2^ < 20%). A random-effects model was employed in the presence of moderate to high heterogeneity, whereas a fixed-effects model was adopted otherwise. All statistical tests were two-sided with a significance level set at P < 0.05. Sensitivity analyses using the leave-one-out method were performed to estimate the stability of the results. Subgroup analyses were performed to investigate possible causes of heterogeneity among study results. Begg’s and Egger’s tests were implemented to assess publication bias, with no significant bias inferred if P > 0.05.

## 3 Results

### 3.1 Search results

**Figure 1:**
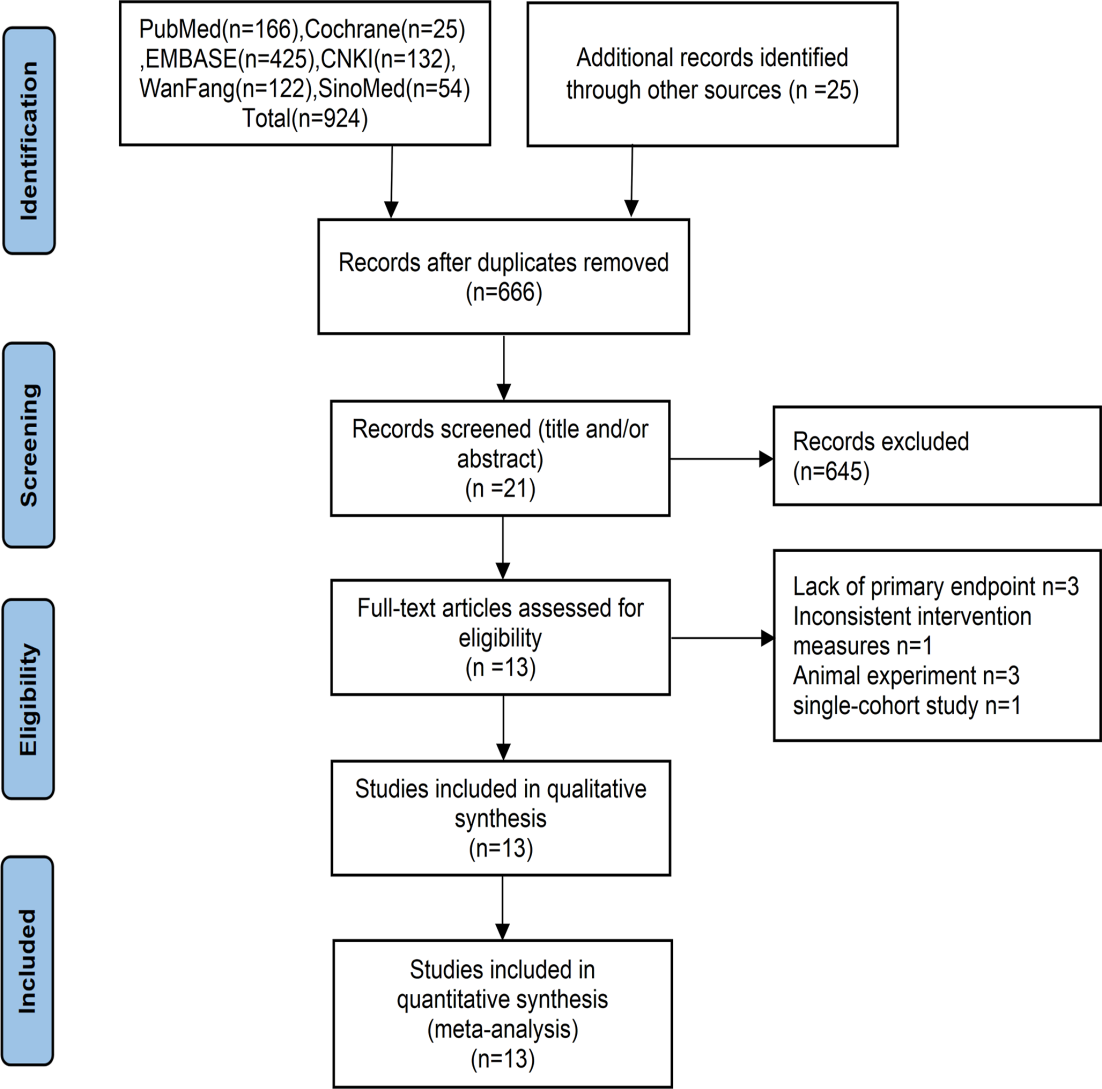
Flow diagram of study selection progress for the systematic review and meta-analyses.

Initially, 949 relevant articles were retrieved from the database, resulting in 666 studies after duplicates were removed. Following review of titles and abstracts, 645 studies were excluded based on the exclusion criteria. Of the remaining studies, 8 studies were deleted due to not meeting the inclusion criteria through full-text reading. Ultimately, 13 studies were identified as eligible for inclusion (**Figure 1**), since Morita’s study is divided into two parts, we will uniformly refer to the number of studies as 14 in the subsequent text.

### 3.2 Characteristics of included studies

As the summary characteristics shown in **Table 1**, the included studies were published between 2020 and 2024. A total of 3,142 patients with cancer were enrolled, including 587 receiving probiotics and 2,555 who did not. Among the 14 studies, 10 studies were retrospective, 4 studies were prospective, in which 2 studies were RCT. 7 studies were conducted in Japan, 3 in the United States, 3 in China, and 1 in the Czech Republic. In Morita’s study, survival data from two cohort studies with different immunotherapy regimens were analyzed separately, which we have indicated in **Table 1**. 5 studies reported the relationship between probiotics use and OS/PFS in cancer patients exposed to antibiotic and receiving ICIs. Additionally, **Table 1** presents the quality evaluation of included studies. 9 articles were recognized as high quality, scoring between 7 and 8 points; 3 were rated as moderate quality with 6 points; both RCTs were assessed as low risk.

**Table 1:** Baseline characteristics of included studies.

Author	year	Study region	Study period	Study type	Cancer type	Patients with/without probiotics			Types of ICIs treatment	Outcome	Antibiotic related analysis	Quality
						Number of patients	Median/ mean age	Male				
Svaton (29)	2020	The Czech Republic	2015-2019	Retrospective	NSCLC	6/218	67	133	Nivo	OS; PFS	NO	6
Tomita (30)	2020	Japan	01/2016-05/2019	Retrospective	NSCLC	39/79	68/67	33/66	Nivo/Pembro/Atezo mono or combination therapy	OS; PFS; ORR; DCR	Yes	8
Miura (31)	2021	Japan	01/2016-07/2018	Retrospective	NSCLC	14/286	65	226	Nivo/Pembro mono	OS; ORR	NO	6
Spencer (38)	2021	United Statea	04/2015-01/2019	Retrospective	Melanoma	49/109	64/64	27/67	Anti-PD1/Anti-CTLA4 mono or Anti-CTLA4 + Anti-PD1	PFS; DCR	NO	7
Takada (32)	2021	Japan	01/2016-09/2018	Retrospective	NSCLC	32/262	67	25/208	Nivo/Pembro mono	OS; PFS	Yes	8
Dizman(39)	2022	United States	04/2019-12/2020	Prospective	mRCC	19/10	66/64	13/8	Nivo + Ipi	PFS; ORR; DCR	NO	Low risk
Takada (33)	2022	Japan	01/2016-09/2018	Retrospective	NSCLC	32/261	NR	233	Nivo/Pembro mono	OS; PFS	Yes	8
Tomita (34)	2023	Japan	01/2019-8/2022	Retrospective	NSCLC	45/55	67/66	33/39	Pembro/Atezo+CT or Atezo +Ais+CT or Nivo+Ipi+CT	OS;PFS	Yes	8
Ebrahimi (25)	2024	United States	11/2021-03/2023	Prospective	mRCC	20/10	68/60	15/5	Nivo + Cabozantinib	PFS; ORR; DCR	NO	Low risk
Luo (35)	2024	China	03/2019-09/2022	Prospective	NSCLC	11/63	58/54	4/24	Anti-PD-1/L1 + CT/Ais or Anti-PD-1/L1 + CT + Ais	OS; PFS; ORR	Yes	8
Morita-ID (36)	2024	Japan	12/2015-05/2018	Retrospective	NSCLC	93/389	68/69	68/312	ICIs mono	OS; PFS; ORR; DCR	NO	8
Morita-ICD (36)	2024	Japan	12/2018-12/2020	Retrospective	NSCLC	77/368	68/69	19/61	ICIs + CT	OS; PFS; ORR; DCR	NO	8
Tong (40)	2024	China	06/2021-12/2022	Prospective	lung cancer	71/182	60/63	59/153	Anti-PD-1/L1 + CT/CRT/Ais or Anti-PD-1/L1 + CT + Ais or Anti-PD-1/L1 + cell therapy	PFS; ORR; DCR	NO	7
Wang (37)	2024	China	03/2019-07/2022	Retrospective	HCC;CRC;GC	79/263	NA	NA	Anti-PD-1 + Ais	OS; PFS; ORR	NO	6

ID, Immunotherapy Database; ICD, Immunochemotherapy Database; NSCLC, non-small cell lung cancer; Nivo, Nivolumab; Pembro, Pembrolizumab; Atezo, Atezolizumab; Ipi, ipilimumab; CT, chemotherapy; CRT, chemoradiotherapy; Ais, Anti-angiogenesis; ICIs, immue checkpoint inhibitors; NA, not available; OS, overall survival; PFS, progression free survival; ORR, objective response rate; DCR, disease control rate; mono, monotherapy.

Summary of details regarding the use of probiotics as shown in **Table 2**. Nine studies clearly documented the types of probiotics, the classification of probiotics strains including Lactobacillus (Lactobacillus, Lactobacillus acidophilus-B (LAC-B), Bio-Three Tablets), Bifidobacterium (Bifidobacterium, BIOFERMIN, Bio-Three Tablets), Streptococcus faecalis (BIOFERMIN-R), Butyric acid bacteria (Clostridium butyricum (CBM588), etc.). Only one study reported that the sources of probiotics included supplements, foods, and other sources with unknown probiotic content. Whereas all other studies solely involved probiotic supplements. In six studies, the duration of probiotic therapy was documented, revealing a median range of 7.5 days to 13.3 months, in two RCTs and one prospective real-world study, the regimen involved continuously receiving probiotics until the occurrence of disease progression, unacceptable toxicity, or reaching the study endpoints.

**Table 2:** Summary of details of the use of probiotics.

Author, year	Details of the use of probiotics					
	Types	Classification of probiotics strains	Pathways of supplementation	Usage duration (median duration)	Time window of use	Indications
Svaton (2020) (29)	Lactobacillus	Lactobacillus	Probiotic supplement	7.5 days	Within 1 month before initiating and 1 month after ICIs	NA
Tomita (2020) (30)	CBM588	Clostridium butyricum	Probiotic supplement	4 months (3 days-28 months)	6 months before initiating and/or concurrently with ICIs	Diarrhea/Constipation/Antibiotics-associated dysbiosis/Immune-related enterocolitis/Non-specific abdominal symptoms
Miura (2021) (31)	Bifidobacterium; Antibiotics-resistant lactic acid bacteria (BIOFERMIN-R); Butyric acid bacteria	Bifidobacterium Streptococcus faecalis; Butyric acid bacteria	Probiotic supplement	NA	Concurrently with ICIs	NA
Spencer (2021) (38)	NA	NA	Probiotic supplement; probiotic food; others of unknown	NA	1 month before initiating ICIs	NA
Takada (2021) (32)	BIOFERMIN; LAC-B; CBM588; Antibiotic-resistant lactic acid bacteria (BIOFERMIN-R)	Bifidobacterium; Clostridium butyricum; Streptococcus faecalis;	Probiotic supplement	4.5 months (1month- >1 year)	Concurrently with ICIs	Diarrhea/Loose stool/Constipation/Other unclear
Dizman (2022) (39)	CBM588	Clostridium butyricum;	Probiotic supplement	Continuously receiving until disease progression or unacceptable toxicity (mPFS=12.7 months)	Concurrently with ICIs	Interventions in RCT
Takada (2022) (33)	BIOFERMIN; LAC-B; CBM588; Antibiotic-resistant lactic acid bacteria (BIOFERMIN-R)	Bifidobacterium; Clostridium butyricum; Streptococcus faecalis;	Probiotic supplement	NA	Concurrently with ICIs	Diarrhea/Loose stool/Constipation/Other unclear
Tomita (2023) (34)	CBM588	Clostridium butyricum;	Probiotic supplement	12 months (7 days-47 months)	3 weeks before initiating and/or concurrently with ICIs	Diarrhea/Constipation/Antibiotics-associated diarrhea/Immune-related enterocolitis/Non-specific abdominal Symptoms/Prophylactic administration with antibiotics
Ebrahimi (2024) (25)	CBM588	Clostridium butyricum;	Probiotic supplement	Continuously receiving until disease progression or unacceptable toxicity	Concurrently with ICIs	Interventions in RCT
Luo (2024) (35)	Bio-Three Tablets	Bifidobacterium; Lactobacillus	Probiotic supplement	NA	2 months before initiating and/or concurrently with ICIs	NA
Morita-ID (2024) (36)	CBM588; other	Clostridium butyricum; other	Probiotic supplement	NA	Before initiating or concurrently with ICIs	NA
Morita-ICD (2024) (36)	NA		Probiotic supplement	NA	Before initiating or concurrently with ICIs	NA
Tong (2024) (40)	NA	NA	Probiotic supplement	Continuously receiving until study endpoint (e.g., tumor progression) (mPFS=13.3 months)	Concurrently with ICIs	Patient’s personal choice
Wang (2024) (37)	NA	NA	Probiotic supplement	NA	1 months before initiating and/or concurrently with ICIs	NA

NA, not available; CBM 588, Clostridium butyricum MIYAIRI 588; LAC-B, Lactobacillus acidophilus-B; ICIs, immue checkpoint inhibitors; CRT, chemoradiotherapy; e.g., exempli gratia.

### 3.3 Overall survival

**Figure 2:**
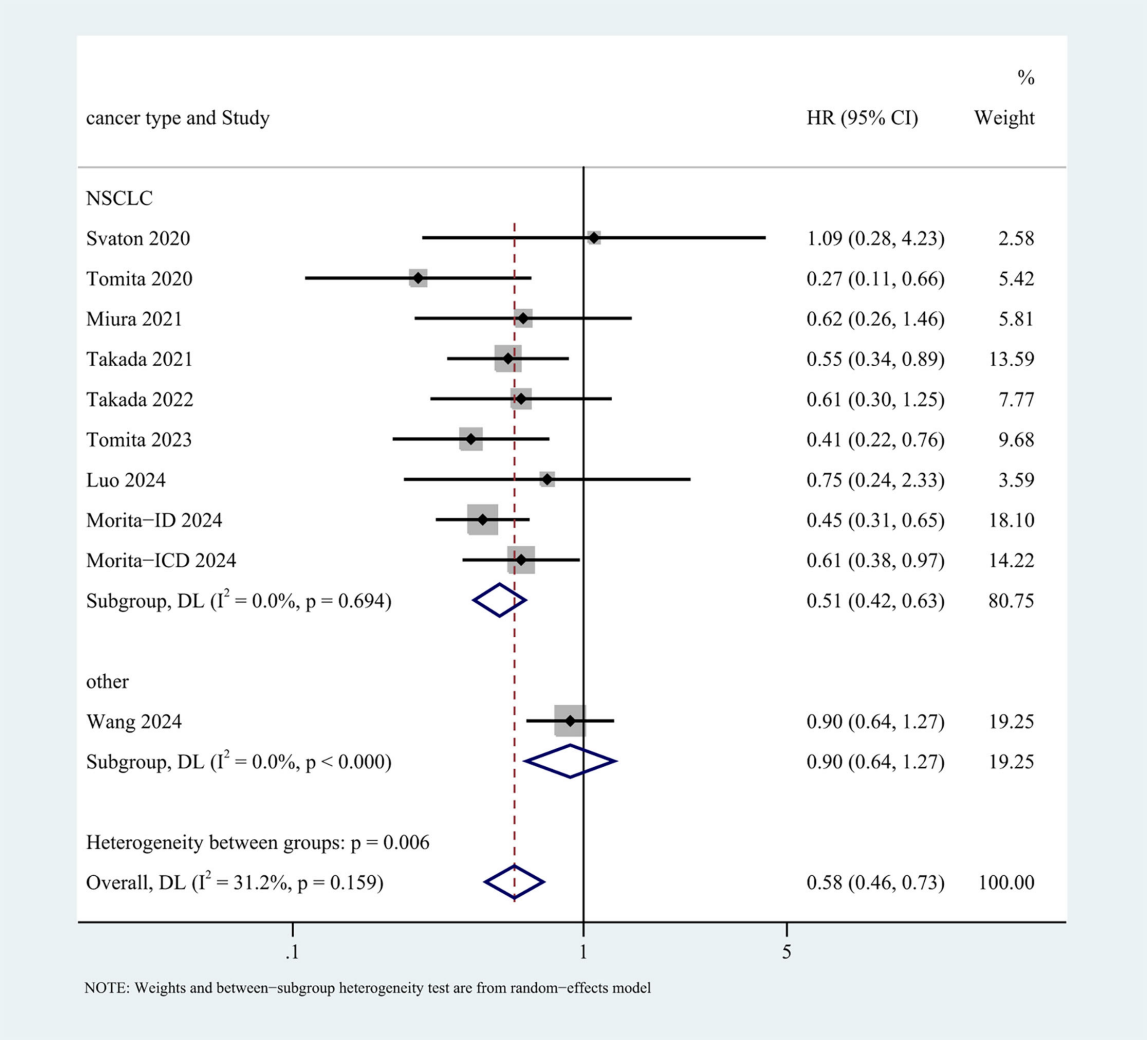
Forest plot of the efficacy of probiotics in overall survival (OS) data.

10 studies (29–37) with a total of 2672 participants (428 receiving probiotics versus 2244 not receiving probiotics) reported data on OS, a random-effects model was employed owing to the moderate heterogeneity among studies (I^2^ = 31.2%). The results revealed that probiotics markedly prolonged OS (HR: 0.58, 95% CI: 0.46-0.73, p < 0.001) (**Figure 2**).

### 3.4 Progression-free survival

**Figure 3:**
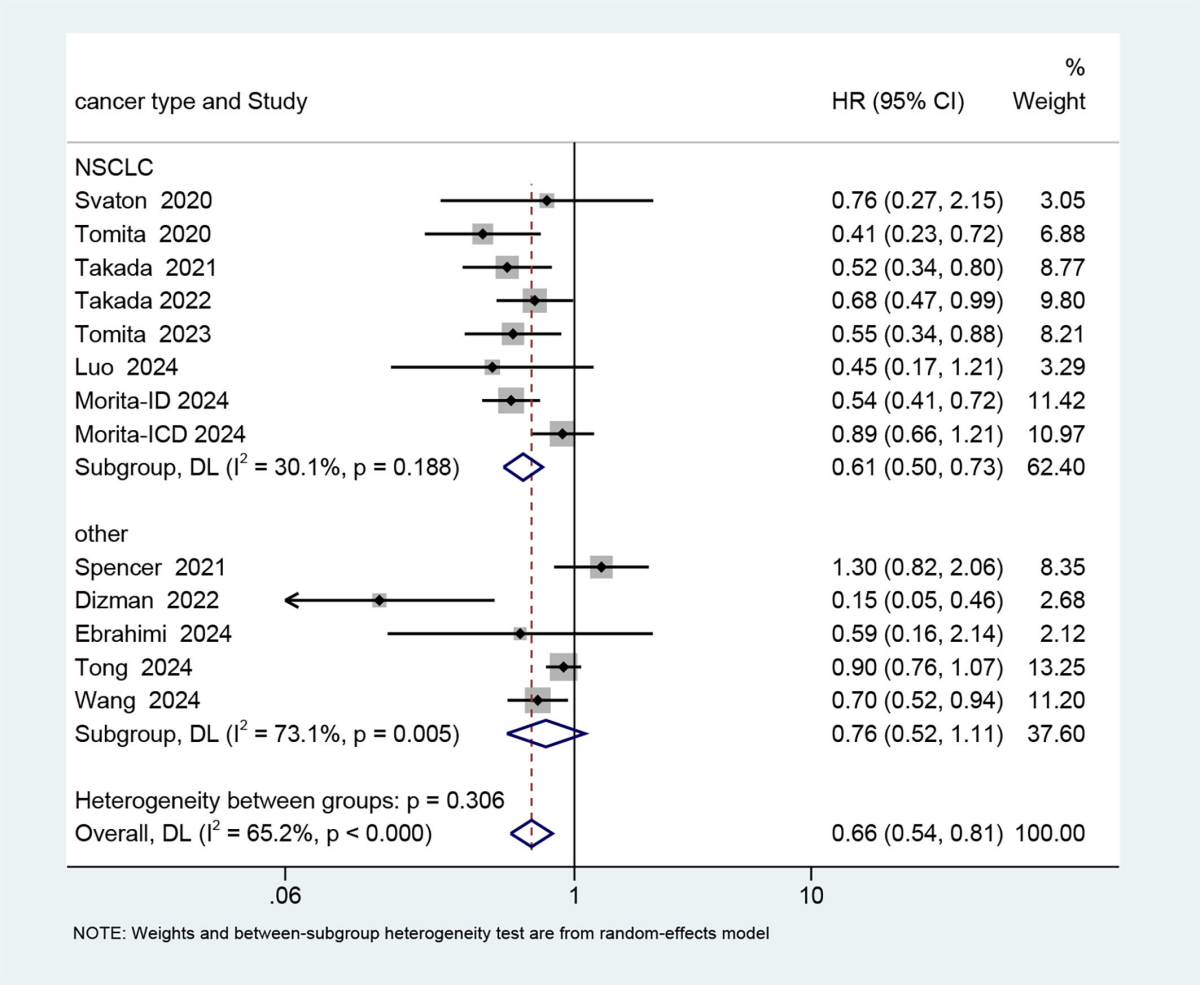
Forest plot of the efficacy of probiotics in progression-free survival (PFS) data.

13 studies (25, 29, 30, 32–40), encompassing 2,842 participants (573 receiving probiotics versus 2269 not receiving probiotics), were included in the meta-analysis of PFS. A random-effects model was chosen considering the high heterogeneity (I^2^ = 65.2%). The findings indicated that probiotics treatment was associated with a reduced risk of poor PFS in cancer patients treated with ICIs (HR: 0.66, 95% CI: 0.54-0.81, p < 0.001) (**Figure 3**).

### 3.5 Subgroup analysis of OS and PFS

To explore possible factors of heterogeneity among OS and PFS, subgroup analyses were conducted base on cancer type, types of probiotics, immunotherapy treatments, ethnic backgrounds, and study types (**Supplementary Tables S2** and **S3**). None of above factors were statistically significant contributors to heterogeneity in PFS subgroup analyses. The observed differences in OS across patients stratified by cancer type (P=0.006) and ethnic backgrounds (P=0.011) reached statistical significance, but the subgroups of “other cancer” and “white” were included only in one study each.

### 3.6 OS and PFS in NSCLC with antibiotic exposure

**Figure 4:**
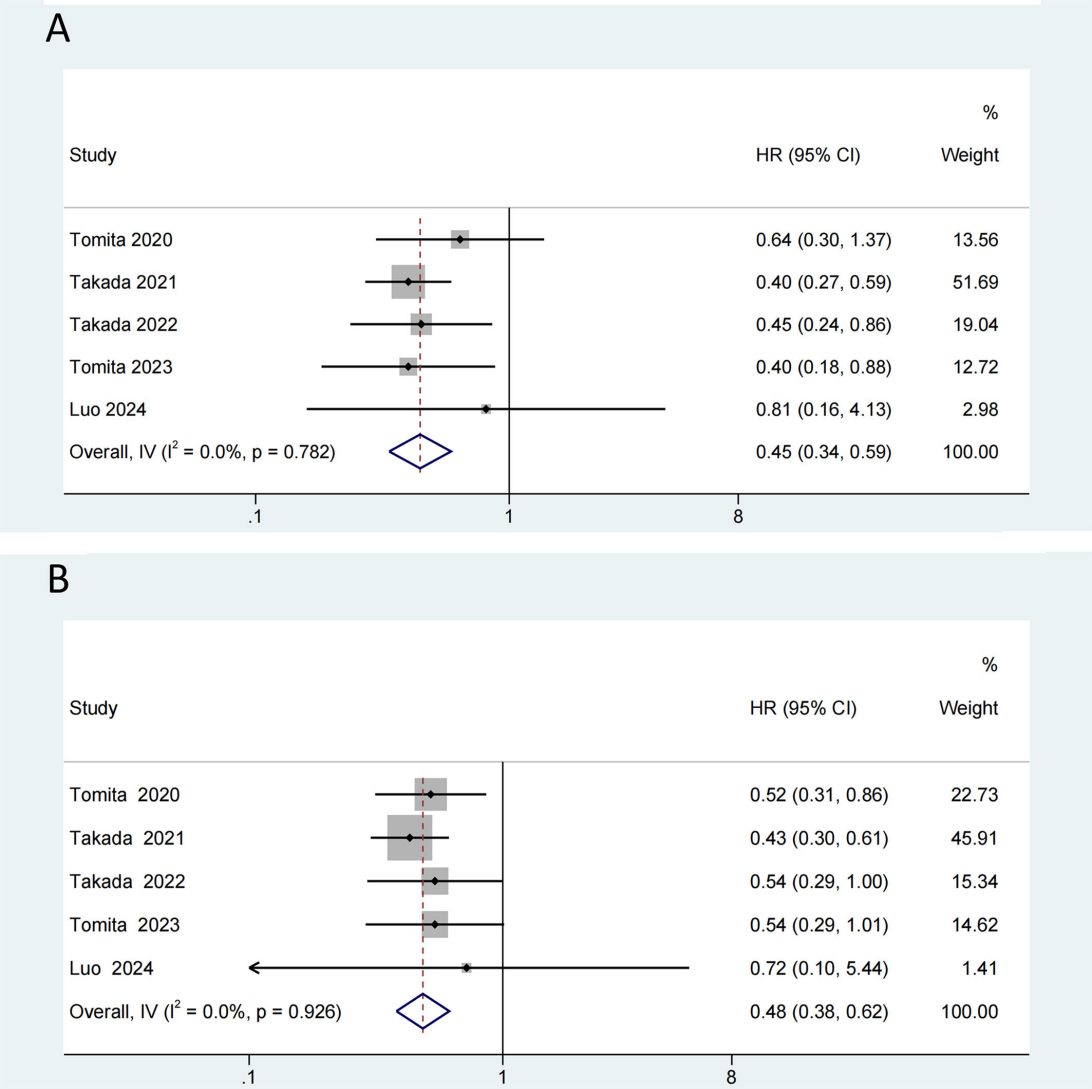
Forest plot of the efficacy of probiotics in overall survival (OS) **(A)** and progression-free survival (PFS) **(B)** in non-small cell lung cancer (NSCLC) with antibiotic exposure.

Prior studies have shown that antibiotic usage diminishes the response to ICIs. Among the 14 studies included in our analysis, 5 studies (30, 32–35), all of which were NSCLC studies, assessed OS and PFS of populations exposed to antibiotics. Our meta-analysis suggested that probiotics could counteract the deterioration in OS caused by antibiotic use (I^2^ = 0.0%; HR=0.45, 95% CI: 0.34-0.59, p < 0.001) (**Figure 4A**). Consistent with the findings for OS, probiotics was significantly associated with better PFS in NSCLC receiving antibiotics (I^2^ = 0.0%; HR=0.48, 95% CI: 0.38-0.62, p < 0.001) (**Figure 4B**).

### 3.7 Objective response rate

**Figure 5:**
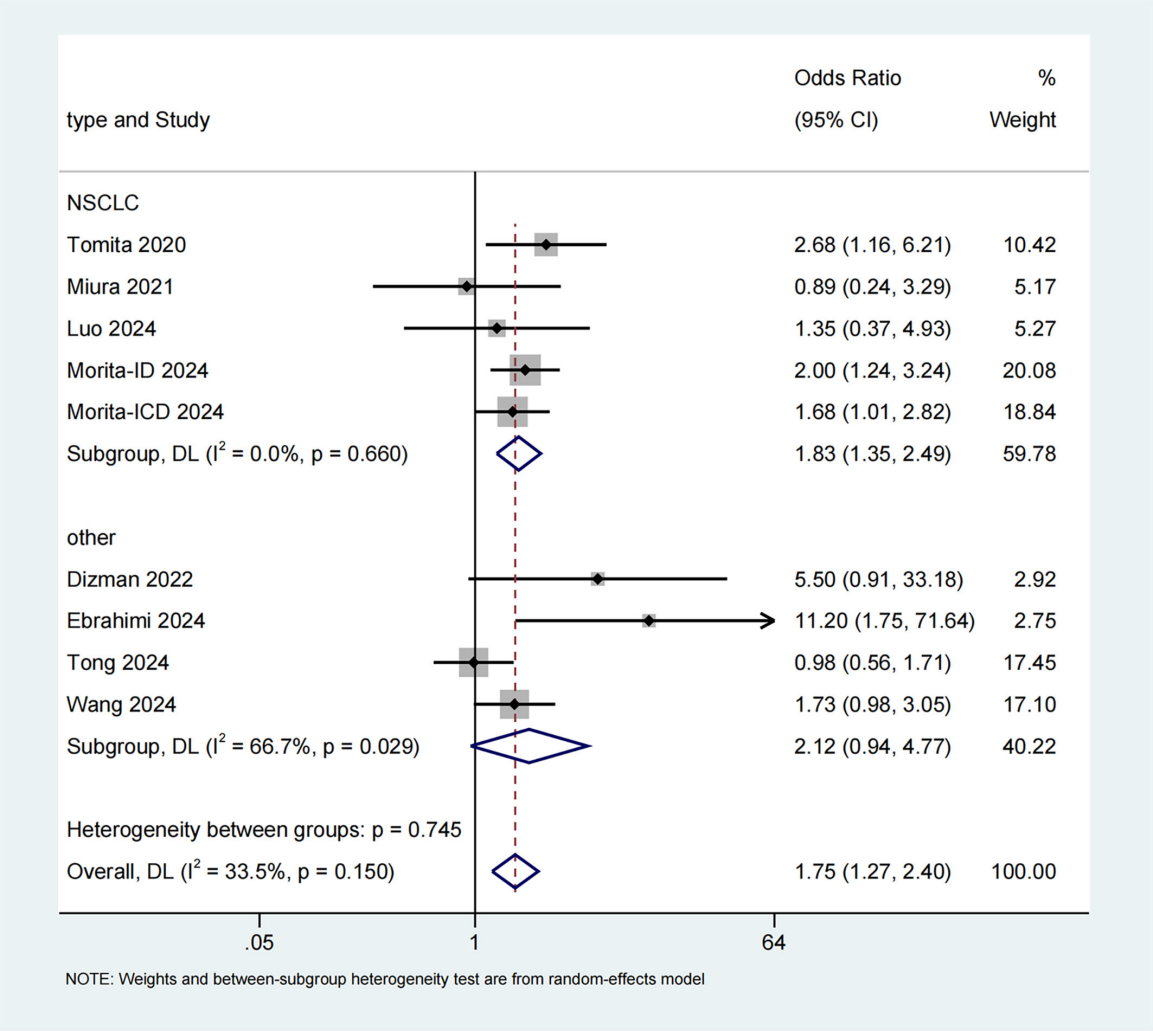
Forest plot of the efficacy of probiotics in objective response rate (ORR) data.

The link between probiotics and ORR was analyzed based on data from 9 studies (25, 30, 31, 35–37, 39, 40). A random-effects model was applied in light of the presence of moderate heterogeneity (I^2^ = 33.5%). Our meta-analysis demonstrated a substantial enhancement in ORR for patients who consumed probiotics (OR=1.75, 95% CI: 1.27-2.40, p = 0.001) (**Figure 5**). Subgroup analysis showed that the findings in NSCLC were consistent with the aforementioned results (I^2^ = 0.0%; OR=1.83, 95% CI: 1.35-2.49, p < 0.001), an improvement in other cancer types was also noted, albeit not statistically significant (I^2^ = 33.5%; OR=1.75, 95% CI: 1.27-2.40, p = 0.07).

### 3.8 Disease control rate

**Figure 6:**
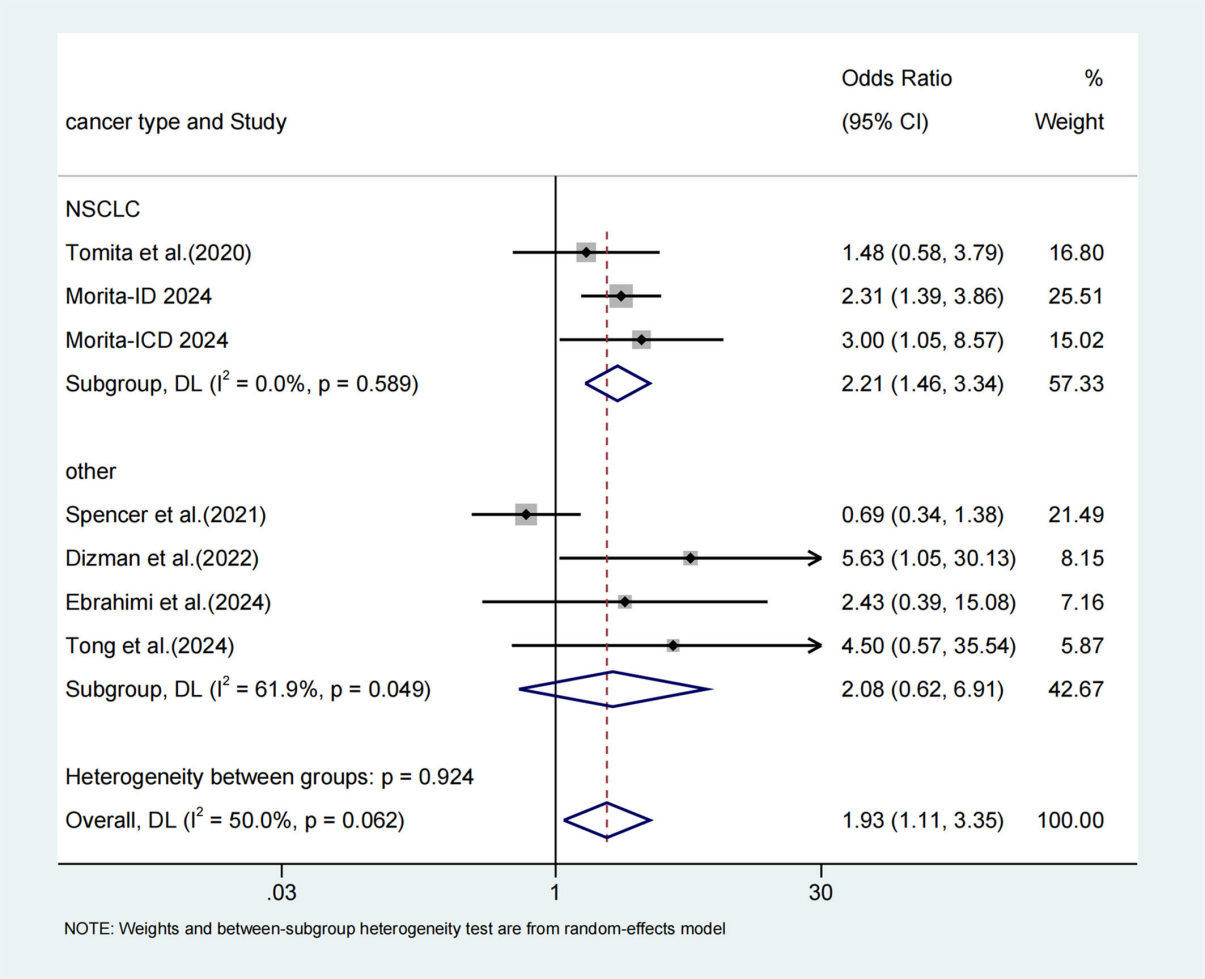
Forest plot of the efficacy of probiotics in disease control rate (DCR) data.

7 studies (25, 30, 36, 38–40) recorded the DCR, a random-effects model was applied because of the high-level heterogeneity (I^2^ = 50.0%). The administration of probiotics was correlated with a better DCR (OR: 1.93, 95% CI: 1.11-3.35, p = 0.002) (**Figure 6**). Subgroup analysis demonstrated that probiotics prominently improved DCR in NSCLC treated with ICIs (I^2^ = 0.0%; OR=2.21, 95% CI: 1.46-3.34, p < 0.001), but without a significant effect on the DCR in other type of tumors (I^2^ = 61.9%; OR=2.08, 95% CI: 0.62-6.91, p = 0.233).

### 3.9 Publication bias and sensitive analysis

**Figure 7:**
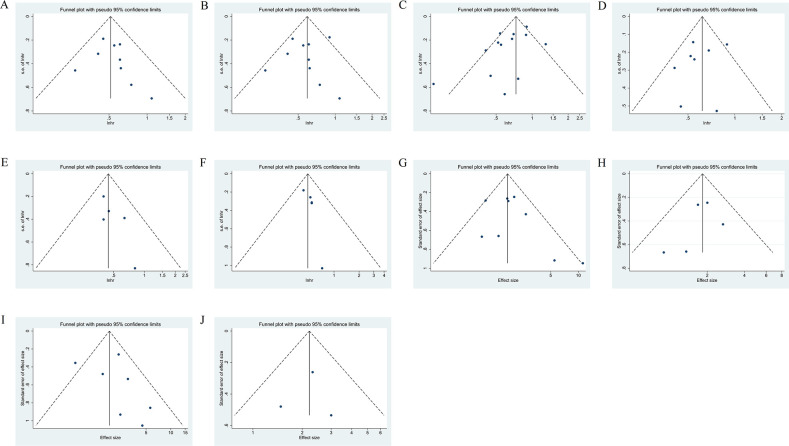
Funnel plot of the HRs for OS **(A)**, OS in NSCLC subgroup **(B)**, PFS **(C)**, PFS in NSCLC subgroup **(D)**, OS in NSCLC with antibiotic exposure **(E)**, PFS in NSCLC with antibiotic exposure **(F)**, and funnel plot of the OR for ORR **(G)**, ORR in NSCLC subgroup **(H)**, DCR **(I)**, DCR in NSCLC subgroup **(J)**. HRs, hazard ratios; OS, Overall survival; PFS, progression-free survival, NSCLC, non-small cell lung cancer; OR, odds ratio; ORR, objective response rate, DCR, disease control rate.

The funnel plot analyses of the result above are presented in **Figure 7**, the Begg’s and Egger’s tests showed no publication bias in the meta-analysis (**Supplementary Table S4**). Moreover, sensitivity analyses confirmed that no individual study significantly affected the pooled results (**Supplementary Figures S1A–J**), suggesting that the results of this meta-analysis are relatively credible and stable.

## 4 Discussion

### 4.1 Principal findings

This study is an update to previous meta-analyses in light of the increasing of newly published data, meanwhile, to our knowledge, it is the first to examine the impact of probiotics supplementation on the survival benefits of immunotherapy among cancer patients with a background of antibiotic use. Most of the retrospective studies included in this analysis collected probiotics usage information through medical record reviews and patient self-reports, however, the presence of unmeasured confounding variables may introduce bias into the findings, necessitating a cautious interpretation of the results. Nonetheless, our meta-analysis results show the same impact trend as those of two RCTs, indicating a certain degree of credibility and value. In this meta-analysis, our results suggest that that the use of probiotics can improve the response rate to tumor immunotherapy, prolong OS and PFS, and elevate tumor remission rate. Furthermore, this effect appears to be particularly pronounced in individuals exposed to antibiotics, indicating that probiotics may help mitigate the detrimental effects of antibiotics on immunotherapy. The sensitivity analysis confirms the robustness of our findings. This study furnishes strong evidence suggesting that probiotics supplementation could serve as a potential therapeutic strategy to enhance immunotherapy efficacy and decrease the incidence of resistance.

The adult human intestinal microbiota, which is widely recognized as the “second genome” of humans, plays a crucial role in human health. The interaction between the host and the microbiota forms a complex and interwoven network (11). Evidence from the past decade has indicated that the gut microbiota and its metabolites are critical factors in modulating the effectiveness and toxicity of cancer immunotherapy (41). Staffas et al. (42) discovered that the depletion of the intestinal microbiota significantly decreased lymphocyte and neutrophil counts. The gut microbiota of patients who respond to immunotherapy differed significantly from those who do not in terms of abundance, diversity, and microbial community structure (12, 43, 44). In 2015, Science published two landmark studies concurrently, which respectively discovered that Bifidobacteria and Bacteroides fragilis, acting as potential probiotics, could boost the anti-tumor efficacy of ICIs (43, 44). Conversely, germ-free mice displayed a compromised immune response, however, the fecal microbiota transplantation (FMT) from responsive patients could restore the immune response. Manipulating the composition of the gut microbiota could be a promising approach to improve the therapeutic outcomes of cancer immunotherapy, including probiotics, prebiotics, FMT, engineered microbial products and diet interventions (41).

Probiotics, characterized as “viable microorganisms” (45), may potentially improve the efficacy of ICIs by increasing gut microbial diversity and promoting a beneficial immune environment when ingested in sufficient quantities. Supplementation with Bifidobacterium has been demonstrated to be crucial in improving ICIs efficacy in animal experiments conducted by Sivan et al. (43). and Dizman et al. (39). A multicenter and retrospective study found that the use of probiotics was correlated with favorable clinical outcomes in patients with advanced or recurrent NSCLC undergoing anti-PD-1 monotherapy. CBM588, a bifidogenic live bacterial product, demonstrated a similar clinical effect trend in a randomized phase 1 trial (25). Another study validated probiotics may become an effective monotherapy for cancer, where a blend of four Clostridiales species demonstrating antitumor effects by activating CD8+ T cells and enhancing tumor immunogenicity (46).

Probiotics demonstrate clinical effectiveness in the treatment of various tumors (47). In the subgroup analysis of OS in this study, except for one study that involved patients with advanced digestive tract cancer, all other studies were conducted on patients with NSCLC. Probiotics have the potential to significantly prolong the OS and PFS in NSCLC undergoing immunotherapy. Characteristics such as ethnicity, race and lifestyle factors (e.g., diet) can influence the composition of the gut microbiota (48–50), however, whether these factors also affect the immunomodulatory effects of probiotics remains inconclusive. Research have shown that a Mediterranean diet or a high-fiber diet can improve the response to tumor immunotherapy (38, 51). Due to the partial absence of information in the original studies included in our meta-analysis, we only performed subgroup analysis base on ethnicity, actually, the dietary habits of different ethnic groups largely vary. Significant differences were observed in OS among the groups in our study, but not in PFS, suggesting that the Japanese population might be more likely to benefit from probiotic treatment, thereby extending OS. Nevertheless, given the significant disparity in the number of studies among the groups, those conclusions should be approached with caution. Similarly, in the study by de Moraes FCA, no definitive conclusions were reached (52).

Morita et al. (36) compared probiotics, including the spore-forming bacterium CBM588, with non-spore-forming bacteria, finding no significant difference in ORR between the these groups. In our study, no significant differences were observed among the probiotic subgroups either. Currently, the publicly available research data lack direct comparisons between various probiotics. Besides the type of probiotics, the abundance of probiotics and the combination of genera also seem to be important. NSCLC patients with higher abundance of Akkermansia muciniphila showed a worse immunotherapy response than those patients lacking Akkermansia muciniphila (53). Designing reasonable consortia of microorganisms is one of the focuses of future research.

Owing to the majority of studies included in this meta-analysis lacking data on concurrent medications (e.g., corticosteroids, proton pump inhibitors (PPIs), nonsteroidal anti-inflammatory drugs (NSAIDs), statins and metformin), we were unable to conduct further subgroup analysis. For instance, taking PPIs as an example, multiple retrospective studies have showed that PPIs use correlated with a shorter OS in ICIs receivers with melanoma and NSCLC (54, 55). Nonetheless, another retrospective study found that therapeutic PPI use independently prolonged PFS and OS, unlike prophylactic use, which had no such effect (37). The effect may be associated with the timing window of PPI use. But conflicting conclusions still exist among studies, Chen et al. demonstrated that receiving PPI as baseline treatment or 60 days before ICI treatment initiation may potentially compromise the clinical efficacy of ICIs (56).

A profoundly immunosuppressive TME constitutes a significant hindrance to the efficacy of immunotherapy. In the past decade, researchers have gained a deeper understanding of the mechanisms by which microbiota regulate the tumor immune microenvironment and mediate immunotherapy. The mechanisms by which probiotics exert tumor immune regulation include secreting related molecules (e.g., lipopolysaccharide, peptidoglycans, flagellin); translocating to the TME, secondary lymph nodes, or other locations; producing metabolites with immunomodulatory properties (e.g., short-chain fatty acids (SCFAs), metabolites of dietary tryptophan); simulating tumor-specific antigens to induce T-cell cross-reactivity and altering the expression levels of immune checkpoint molecules, among others (57). Akkermansia muciniphila restored the effectiveness of PD-1 blockade in an interleukin-12-dependent manner by increasing the recruitment of CCR9+CXCR3+CD4+ T lymphocytes into mouse tumor beds (12). Bifidobacterium pseudolongum produces inosine, which increases T cell responses in the TME via adenosine A2A receptor (A2AR) signaling, and thereby improving responses to immunotherapies (58). Butyrate, a kind of SCFAs produced by anaerobic bacteria, could inhibit histone deacetylase activity in CD8+ T cells and induce expression of inhibitor of DNA binding 2 (ID2), finally increase the activation of T cells and reduces T cell exhaustion (59). Additionally, the microbiota can modulate immunity by affecting amino acid metabolism. Oral gavage of Lactobacillus reuteri (Lr) to mice effectively inhibit the growth of B16F10 melanoma, Lr can translocate to, colonize, and persist within melanoma. In this environment, Lr releases the dietary tryptophan catabolite indole-3-aldehyde (I3A), which enhances the activity of CD8+ T cells through the activation of the aryl hydrocarbon receptor (AHR) signaling pathway (60).

However, inconsistencies among probiotics studies persist, whether in terms of clinical efficacy or mechanisms of action. The immunomodulatory mechanisms of probiotics on tumors may involve a complex network of multi-target, multi-pathway and bidirectional interactions; further in-depth investigation is needed to understand the effects of microbiota on tumor immunity.

### 4.2 Probiotics weaken the detrimental effects of antibiotics on ICIs

The extensive application of antibiotics in medical practice is a predominant factor contributing to the disruption of the gut microbiome. Antibiotic administration can lead to significant alterations in the microbiome’s community structure, species composition, and metabolic functions (15), resulting in marked reductions in the relative abundance of the phyla Bacteroidetes, Firmicutes, and Actinobacteria (61). The change in microbial biodiversity lead to a diminished capacity for bile acid conversion (manifested by an elevation in primary bile acids) and a decline in carbohydrate fermentation processes (characterized by a reduction in SCFAs) (62, 63). These microbiota-originated metabolites are crucial for the development and homeostasis of immune cells (64).

A meta-analysis of PFS data from 2,208 patients and OS data from 5,560 patients yielded hazard ratios of 1.47 (95% confidence interval [CI]: 1.13–1.90) for PFS and 1.69 (95% CI: 1.25–2.29) for OS, corresponding to a median decrease in OS of 6.7 months (95% CI: 5.1–8.4) (18). The findings indicated a significant reduction in survival among NSCLC patients exposed to antibiotics. An umbrella review encompassing 23 meta-analyses yielded Class II-IV evidence suggesting that antibiotics exert a detrimental effect on the efficacy of ICIs. This effect was most pronounced when antibiotics were administered within 1 month prior to the initiation of immunotherapy, leading to an increased risk of progression disease (PD) in cancer patients (65). Mucosal vascular addressin cell adhesion molecule 1 (MAdCAM1), a critical checkpoint molecule, is expressed in the intestinal lymphatic system. The downregulation of MAdCAM1 induced by antibiotic administration results in the release of a range of T cell populations primed in the colon and imbalance of T cell subtypes. The migration of IL-17-secreting α4β7+ regulatory T cells (Treg) to the tumor-draining lymph nodes (TDLNs) and the tumour suppressed CD8+ T cell responses to anti-PD1 (66). Prior studies have indicated that the administration of CBM588 in NSCLC, especially those concurrently treated with PPIs and/or antibiotics, was associated with enhanced survival outcomes in a cohort undergoing ICIs monotherapy (30, 34).

### 4.3 Comparison with others

Previous meta-analyses and systematic reviews have evaluated the relationship between probiotics and immunotherapy in people with malignant oncology (24, 67). In 2022, Zhang et al. (67) conducted a meta-analysis of 6 trials demonstrated that cancer patients treated with ICIs plus probiotics exhibited prolonged OS (HR: 0.526, 95% CI: 0.341-0.812, p = 0.004) and increased ORR (OR: 2.831, 95% CI: 1.578-5.076, p < 0.001) in multiple types of cancer, but no statistically significant effect was noted on PFS (HR: 0.585, 95% CI: 0.328-1.045, p = 0.070) or DCR (HR: 1.868, 95% CI: 0.890-3.922, p = 0.099). Though subgroup analysis showed that probiotics achieve significantly longer OS and PFS, higher ORR and DCR in NSCLC (P< 0.05), but this evidence is currently limited, as it is based on only 2 or 3 retrospective studies. Another systematic review by Wan et al. (24) published in 2023, in their study, literature prior to February 2022 was searched and ultimately included in 5 retrospective articles, in comparison with the study by Zhang et al., only a new published study by Takada et al. been added. The meta-analysis showed the improvement in OS (HR = 0.50, 95% CI: 0.3-0.85, p = 0.01) and PFS (HR = 0.51, 95% CI: 0.42-0.61, p < 0.01) by probiotics, but no association was found with ORR (OR = 2.11, 95%CI: 0.51-8.65, p = 0.30), which contradicts the previous study. Furthermore, for the same studies co-included in the analysis, there were discrepancies in the data extracted by the researchers of the two meta-analyses. In 2024, latest data from an open-label, randomized, investigator-initiated, phase 1 study were published, showing that the combination of Cabozantinib and nivolumab with bacterial supplementation did not meet the research endpoints of OS and PFS in metastatic renal cell carcinoma (25). Those studies did not perform a comprehensive analysis of the molecular characteristics of the enrolled patients, which may potentially mask the effects of specific factors that could influence the efficacy of immunotherapy.

### 4.4 Strengths and limitations of the study

The strengths of this study include the following: 1) The inclusion of multiple newly published data, including 1 RCT and 2 prospective observational study, which provide improved statistical power, our meta-analysis was based on the largest curated list of studies to date, comprising data from 13 studies; 2) Addressing the question of whether probiotics can mitigate the negative effects of combination antibiotic therapy on ICIs, we provide the first evidence-based support; 3)A summary table provided delineates specifics regarding probiotics, thereby augmenting both transparency and reproducibility; 4) conducting subgroup analyses to address potential confounding factors, thereby enhancing the stability of research findings.

Several inherent limitations should also be considered: 1) The main limitation of this work is the heterogeneity of included studies, our findings primarily based on observational studies that may be subject to bias due to unmeasured and residual confounding factors, such as concurrent medications, dietary habits and drug administration time window; 2) The types of probiotics, duration and stage of their administration, etc all have potential impacts on the research results, we are unable to conduct subgroup analysis based on these variables due to the unavailability of individual patient; 3) A notable discrepancy in patient numbers exists between the probiotics treatment group and the non-probiotics control group.

## 5 Conclusions

Overall, results from this meta-analysis reveal that probiotics use is positively correlated with better OS, PFS, ORR and DCR in cancer patients administrated with ICIs, especially in NSCLC. Probiotics supplementation significantly mitigates the decreased efficacy of ICIs in NSCLC who received antibiotics. Multi-center, larger sample sizes, standardized treatment protocols and prospective designed studies are warranted to validate the aforementioned findings.

## Data Availability

The original contributions presented in the study are included in the article/**Supplementary Material**. Further inquiries can be directed to the corresponding authors.
