# Supplementary material for: Assessing the impact of probiotics on immunotherapy effectiveness and antibiotic-mediated resistance in cancer: a systematic review and meta-analysis
Source: Front Immunol. 2025 Mar 21;16:1538969. doi: 10.3389/fimmu.2025.1538969 (PMC11968366; doi:10.3389/fimmu.2025.1538969)
Supplement: Supplementary file 1 [file DataSheet1.docx]

**Supplementary materials**

**Assessing the impact of probiotics on immunotherapy effectiveness and antibiotic-mediated resistance in cancer A systematic review and meta-analysis**

**Supplemental Table S1. Search strategy.**

**Supplemental Table S2. Analyses of subgroup factors and test for subgroup differences in OS**

**Supplemental Table S3. Analyses of subgroup factors and test for subgroup differences in PFS**

**Supplemental Table S4. The Begg’s and Egger’s tests**

**Supplemental Figure S1A-J. Sensitivity analyses**

**Supplemental Table S1. Search strategy**

PubMed search as an example：

|  | **Search Terms/Phrases** |
| --- | --- |
| #1 | neoplasms[mesh] |
| #2 | (tumors) OR (neoplasia)) OR (neoplasias)) OR (neoplasm)) OR (tumor)) OR (cancer)) OR (cancers)) OR (malignant neoplasm)) OR (malignancy)) OR (malignancies)) OR (malignant neoplasms)) OR (neoplasm, malignant)) OR (neoplasms, malignant)) OR (benign neoplasms)) OR (neoplasms, benign)) OR (neoplasm, benign)) OR (benign neoplasm)) OR (oncology)) OR (carcinoma) |
| #3 | (#1) OR (#2) |
| #4 | immune checkpoint inhibitors[mesh] |
| #5 | (immune checkpoint inhibitors) OR (checkpoint inhibitors, immune) OR (immune checkpoint blockers) OR (checkpoint blockers, immune) OR (immune checkpoint inhibitor) OR (checkpoint inhibitor, immune) OR (CTLA-4 inhibitors) OR (CTLA 4 inhibitors) OR (cytotoxic t-lymphocyte-associated protein 4 inhibitors) OR (cytotoxic t-lymphocyte-associated protein 4 inhibitors) OR (cytotoxic t-lymphocyte-associated protein 4 inhibitor) OR (cytotoxic t lymphocyte associated protein 4 inhibitor) OR (CTLA-4 inhibitor) OR (CTLA 4 inhibitor) OR (PD-1 inhibitors) OR (PD 1 inhibitors) OR (programmed cell death protein 1 inhibitor) OR (programmed cell death protein 1 inhibitors) OR (PD-1 inhibitor) OR (inhibitor, PD-1) OR (PD 1 inhibitor) OR (immune checkpoint blockade) OR (checkpoint blockade, immune) OR (immune checkpoint inhibition) OR (checkpoint inhibition, immune) OR (PD-L1 inhibitors) OR (PD L1 inhibitors) OR (programmed death-ligand 1 inhibitors) OR (programmed death ligand 1 inhibitors) OR (PD-L1 inhibitor) OR (PD L1 inhibitor) OR (PD-1/PD-L1 blockade) OR (blockade, PD-1/PD-L1) OR (PD 1 PD L1 blockade) OR (ICI) OR (ICIs) OR (nivolumab) OR (pembrolizumab) OR (avelumab) OR (atezolizumab) OR (tremelimumab) OR (pidilizumab) OR (durvalumab) OR (lambrolizumab) OR (cemiplimab) OR (ipilimumab) OR (camrelizumab) OR (tislelizumab) OR (sintilimab) |
| #6 | (#4) OR (#5) |
| #7 | probiotics[mesh] |
| #8 | (probiotics) OR (probiotic agent)) OR (probiotic)) OR (live bacterial) |
| #9 | (#7) OR (#8) |
| #10 | (#3) AND (#6) AND (#9) |

**Supplemental Table S2. Analyses of subgroup factors and test for subgroup differences in OS**

| **OS** | **No. Of studies** | **Pooled HR, (95%CI)** | **P pooled** | **I^2^** | **Heterogeneity between subgroups** |
| --- | --- | --- | --- | --- | --- |
| Overall | 10 | 0.58 (0.46-0.73) | P＜0.001 | 31.2% | NA |
| **Cancer types** |  |  |  |  | **P=0.006** |
| NSCLC | 9 | 0.51 (0.42-0.63) | P＜0.001 | 0.0% |  |
| other | 1 | 0.90 (0.64-1.27) | P=0.551 | NA |  |
| **Types of probiotics** |  |  |  |  | **P=0.222** |
| Multiple probiotic | 3 | 0.58 (0.40-0.83) | P=0.003 | 0.0% |  |
| Clostridium butyricum | 2 | 0.36 (0.22-0.60) | P＜0.001 | 0.0% |  |
| Lactobacillus | 1 | 1.09 (0.28-4.23) | P=0.905 | NA |  |
| Unclear/other | 4 | 0.64 (0.44-0.93) | P=0.020 | 58.9% |  |
| **Immunotherapy** |  |  |  |  | **P=0.203** |
| Monotherapy | 6 | 0.50 (0.39-0.64) | P＜0.001 | 0.0% |  |
| Combination therapy | 4 | 0.66 (0.46-0.95) | P=0.026 | 42.7% |  |
| **Ethnic backgrounds** |  |  |  |  | **P=0.011** |
| Japanese | 7 | 0.50 (0.41-0.61) | P＜0.001 | 0.0% |  |
| Chinese | 2 | 0.89 (0.64-1.23) | P=0.475 | 0.0% |  |
| White | 1 | 1.09 (0.28-4.23) | P=0.905 | NA |  |
| **Study types** |  |  |  |  | **P=0.644** |
| Retrospective | 9 | 0.57 (0.45-0.73) | P＜0.001 | 38% |  |
| Prospective | 1 | 0.75 (0.24-2.33) | P=0.619 | NA |  |

OS, overall survival; No., number; HR, hazard ratio; CI, confidence interval; I^2^, I^2^ statistic; NA, not available; NSCLC, non-small cell lung cancer. “Multiple probiotic” refers to a combination that includes bifidobacterium, streptococcus faecalis and butyric acid bacteria.

**Supplemental Table S3. Analyses of subgroup factors and test for subgroup differences in PFS**

| **PFS** | **No. Of studies** | **Pooled HR, (95%CI)** | **P pooled** | **I^2^** | **Heterogeneity between subgroups** |
| --- | --- | --- | --- | --- | --- |
| Overall | 13 | 0.66 (0.54-0.81) | P＜0.001 | 65.2% | NA |
| **Cancer types** |  |  |  |  | **P=0.306** |
| NSCLC | 8 | 0.61 (0.50-0.73) | P＜0.001 | 30.1 |  |
| Other | 5 | 0.76 (0.52-1.11) | P=0.152 | 73.1 |  |
| **Type of probiotics** |  |  |  |  | **P=0.101** |
| Multiple probiotic | 2 | 0.61 (0.46-0.81) | P=0.001 | 0.0 |  |
| Clostridium butyricum | 4 | 0.42 (0.26-0.66) | P＜0.001 | 35.7 |  |
| Lactobacillus | 1 | 0.76 (0.27-2.15) | P=0.612 | NA |  |
| Unclear/other | 6 | 0.79 (0.62-1.00) | P=0.048 | 69.0 |  |
| **Immunotherapy** |  |  |  |  | **P=0.149** |
| Monotherapy | 7 | 0.58 (0.41-0.83) | P=0.002 | 70.1 |  |
| Combination therapy | 6 | 0.78 (0.65-0.93) | P=0.005 | 28.2 |  |
| **Ethnic backgrounds** |  |  |  |  | **P=0.301** |
| Japanese | 6 | 0.61 (0.49-0.75) | P＜0.001 | 47.1 |  |
| Chinese | 3 | 0.78 (0.61-1.01) | P=0.062 | 44.9 |  |
| White | 4 | 0.59 (00.23-1.53) | P=0.275 | 76.5 |  |
| **Study types** |  |  |  |  | **P=0.422** |
| Retrospective | 9 | 0.67 (0.55-0.83) | P＜0.001 | 56.9 |  |
| Prospective | 2 | 0.76 (0.42-1.36) | P=0.356 | 45.8 |  |
| RCT | 2 | 0.29 (0.07-1.09) | P=0.067 | 59.5 |  |

PFS, progression free survival; No., number; HR, hazard ratio; CI, confidence interval; I^2^, I^2^ statistic; NA, not available; NSCLC, non-small cell lung cancer; RCT, randomized controlled trial. “Multiple probiotic” refers to a combination that includes bifidobacterium, streptococcus faecalis and butyric acid bacteria.

**Supplemental Table S4. The Begg’s and Egger’s tests**

| **Outcome** | **Begg’s t** | **Egger’s** | **Subgroup Outcome** | **Begg’s t** | **Egger’s** |
| --- | --- | --- | --- | --- | --- |
| OS | 0.721 | 0.680 | OS in NSCLC subgroup | 0.348 | 0.439 |
| PFS | 0.360 | 0.064 | PFS in NSCLC subgroup | 0.902 | 0.458 |
| OS in NSCLC with antibiotic exposure | 0.462 | 0.143 | - | - | - |
| PFS in NSCLC with antibiotic exposure | 0.086 | 0.091 | - | - | - |
| ORR | 0.348 | 0.280 | ORR in NSCLC subgroup | 0.221 | 0.435 |
| DCR | 0.764 | 0.438 | DCR in NSCLC subgroup | 1.000 | 0.902 |

NSCLC, non-small cell lung cancer; OS, overall survival; PFS, progression free survival; ORR, objective response rate; DCR, disease control rate.

**Supplemental Figure S1A-J. Sensitivity analyses**

Figure S1A. OS


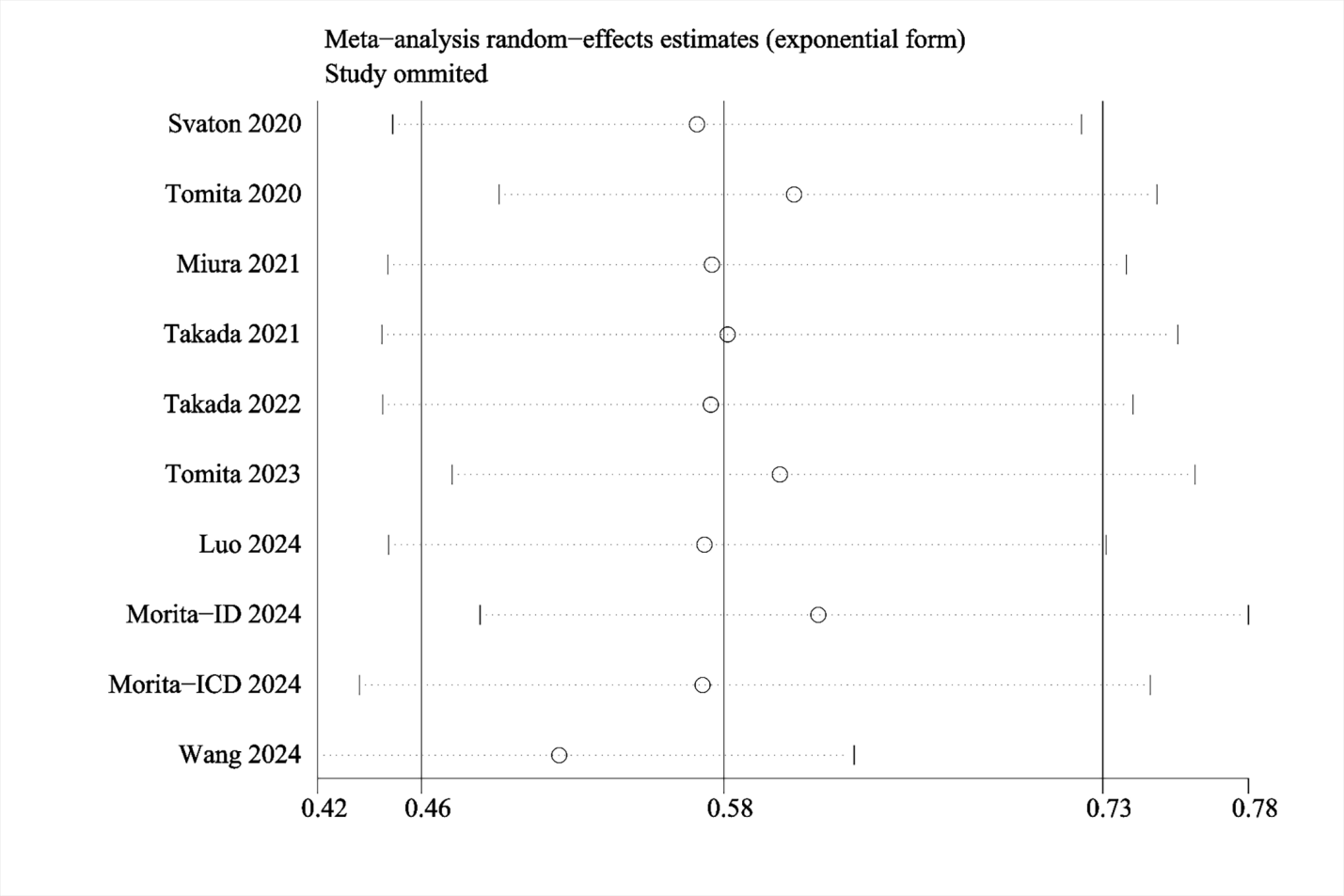


Figure S1B. OS in NSCLC subgroup


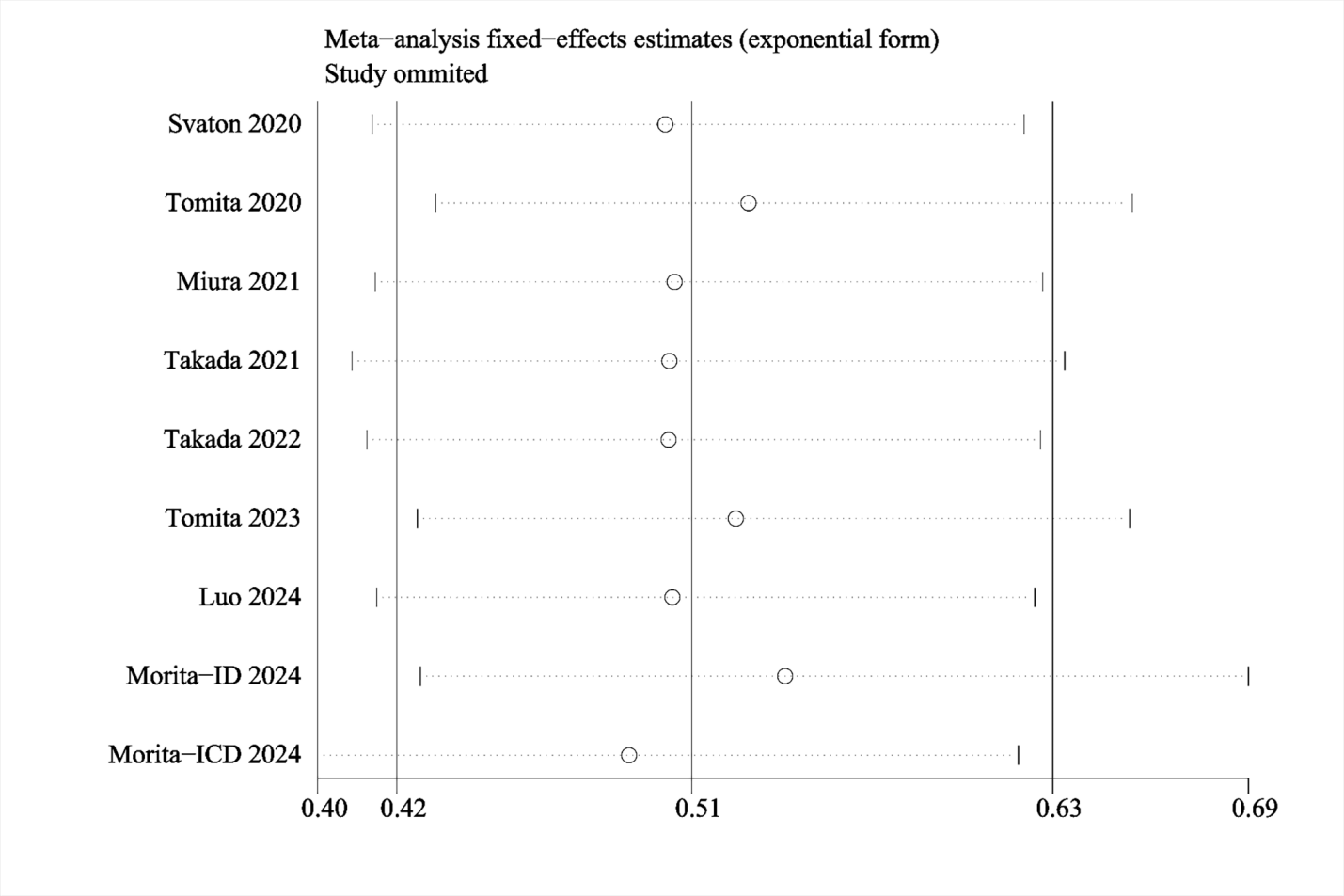


Figure S1C. PFS


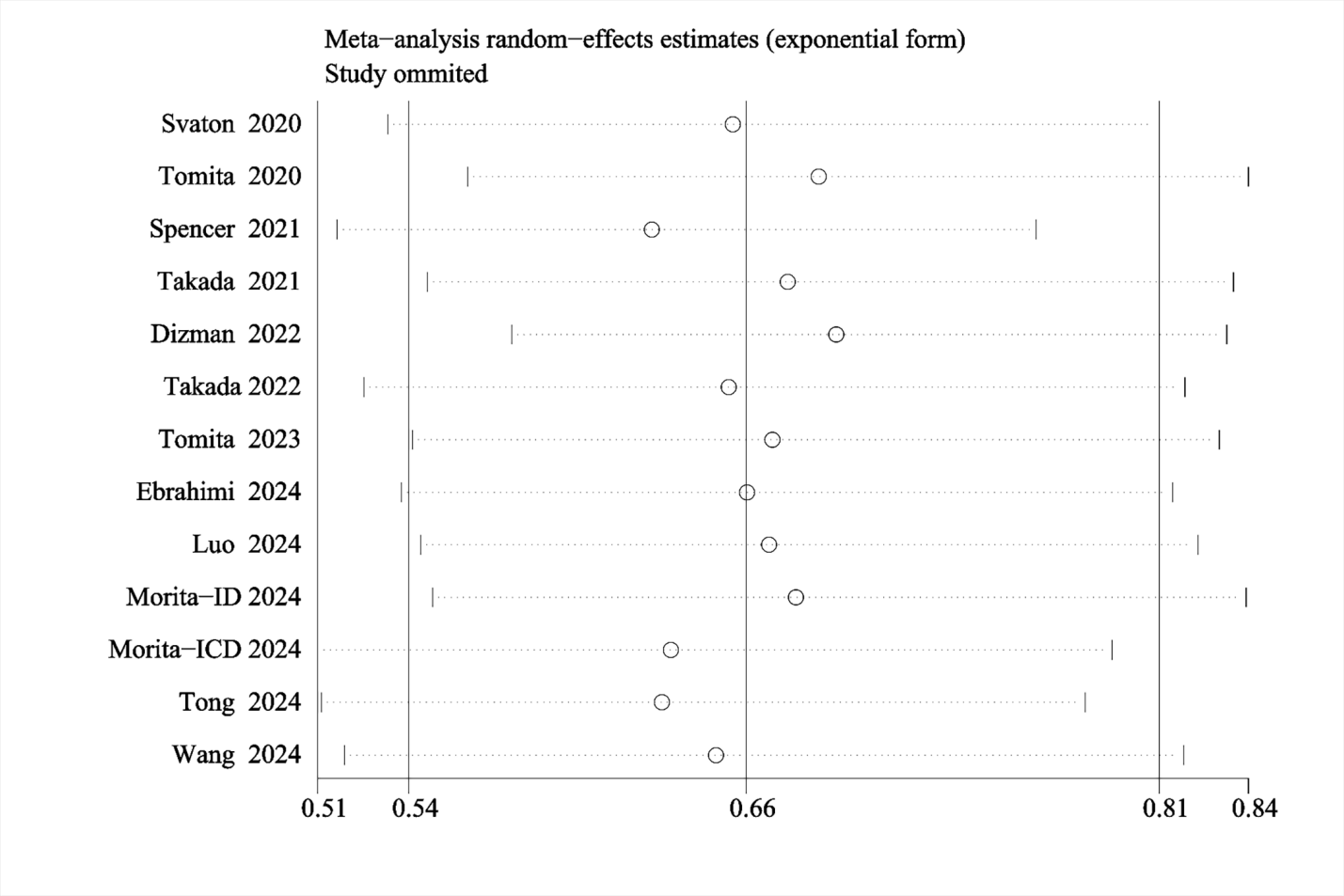


Figure S1D. PFS in NSCLC subgroup


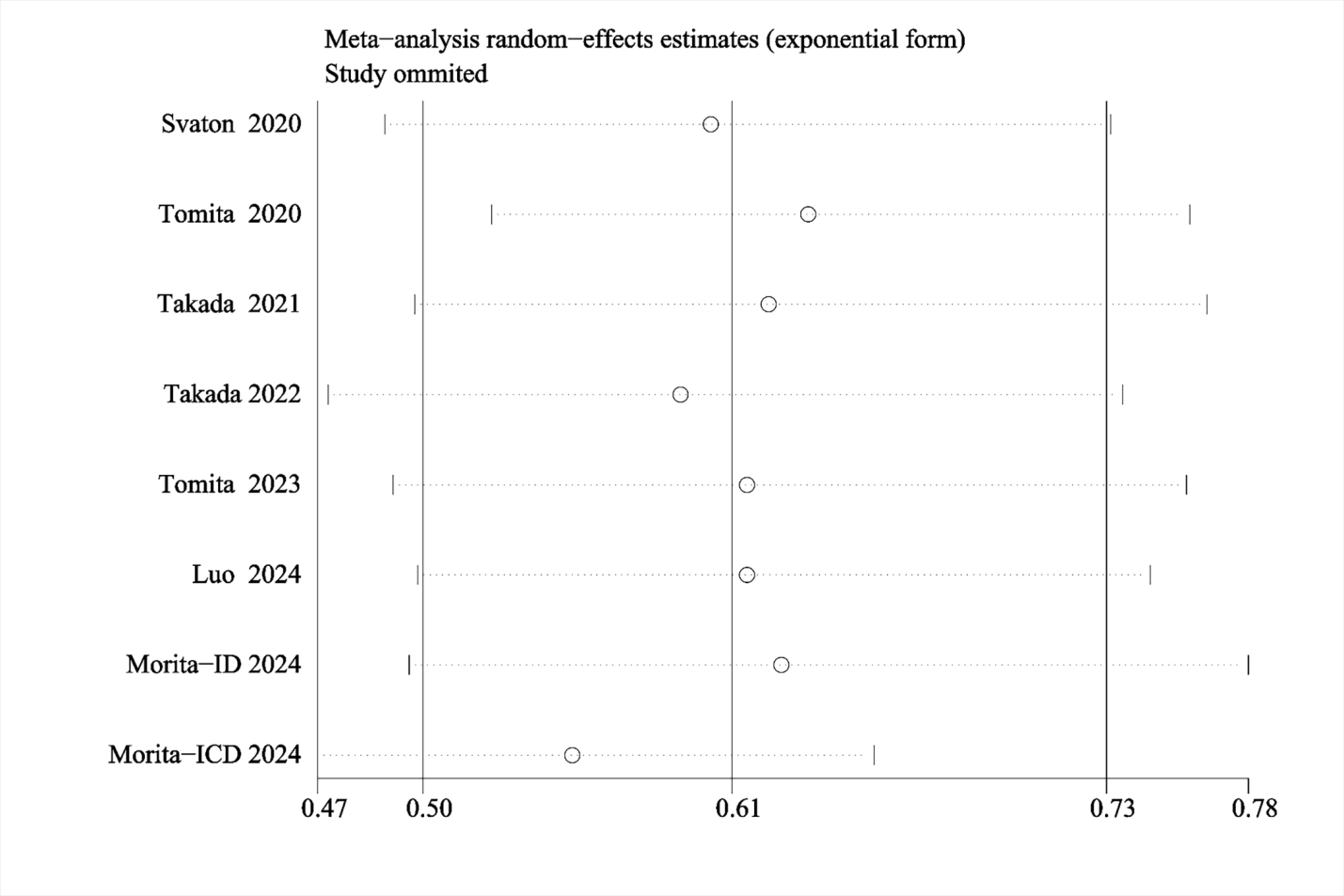


Figure S1E. OS in NSCLC with antibiotic exposure


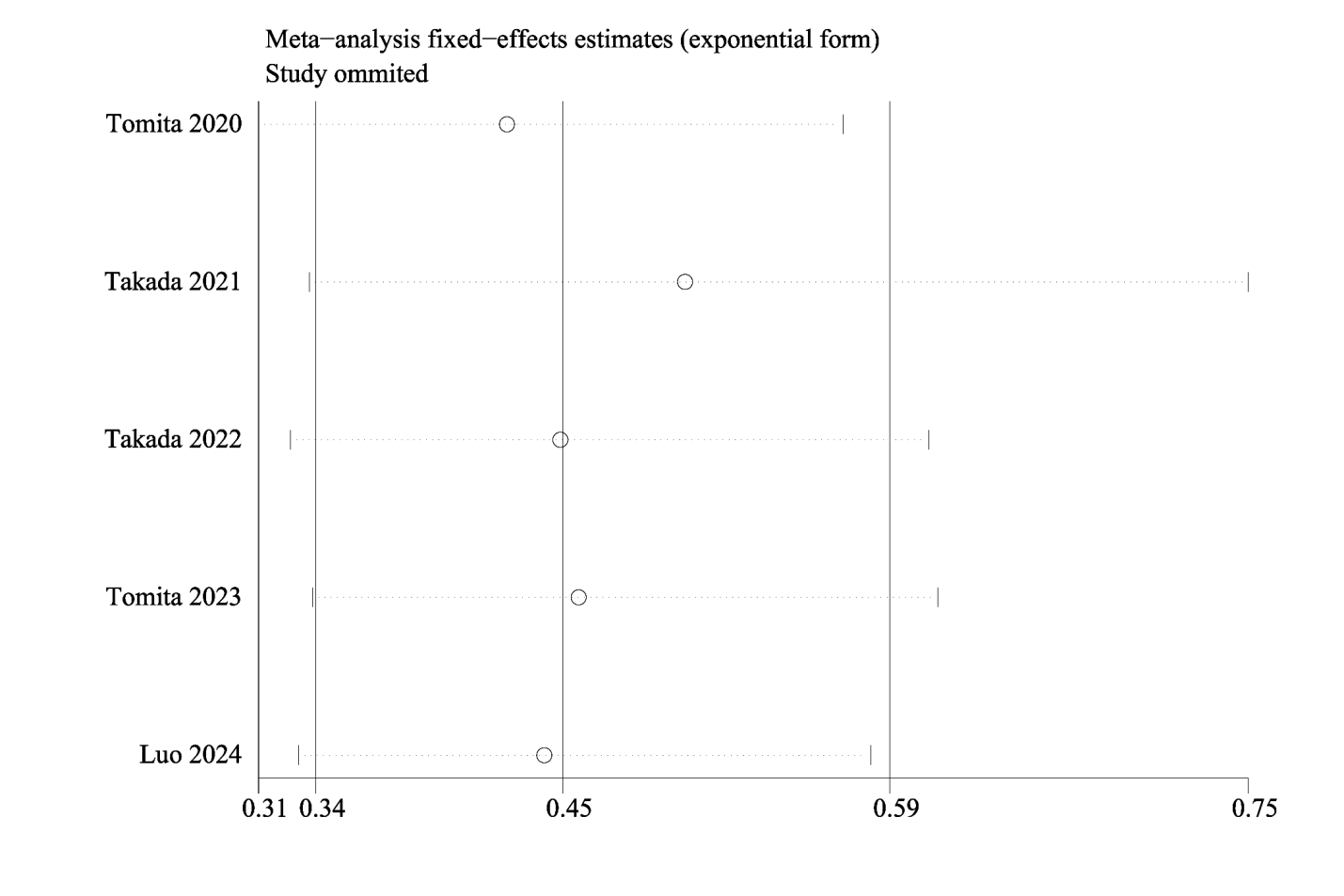


Figure S1F. PFS in NSCLC with antibiotic exposure


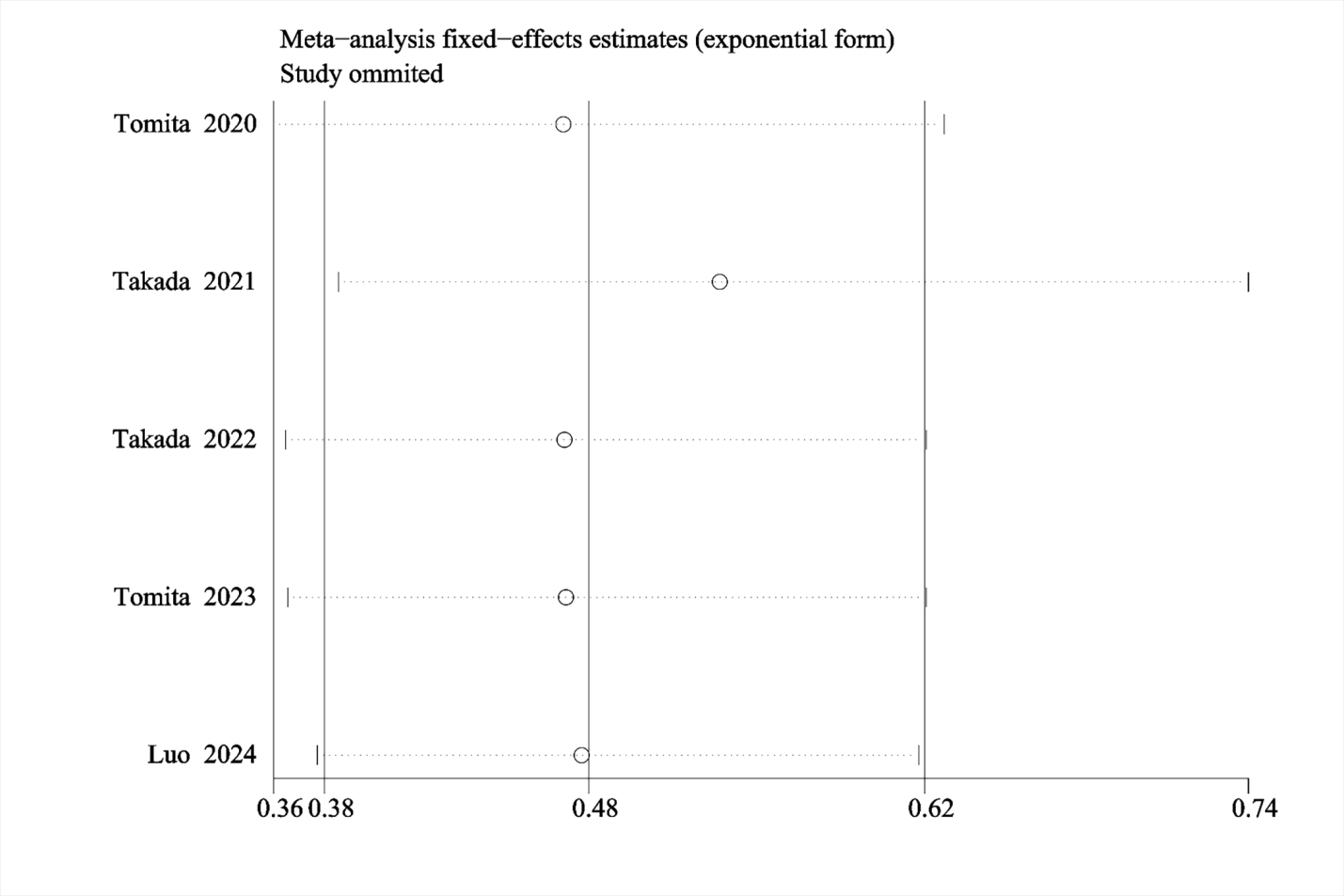


Figure S1G. ORR


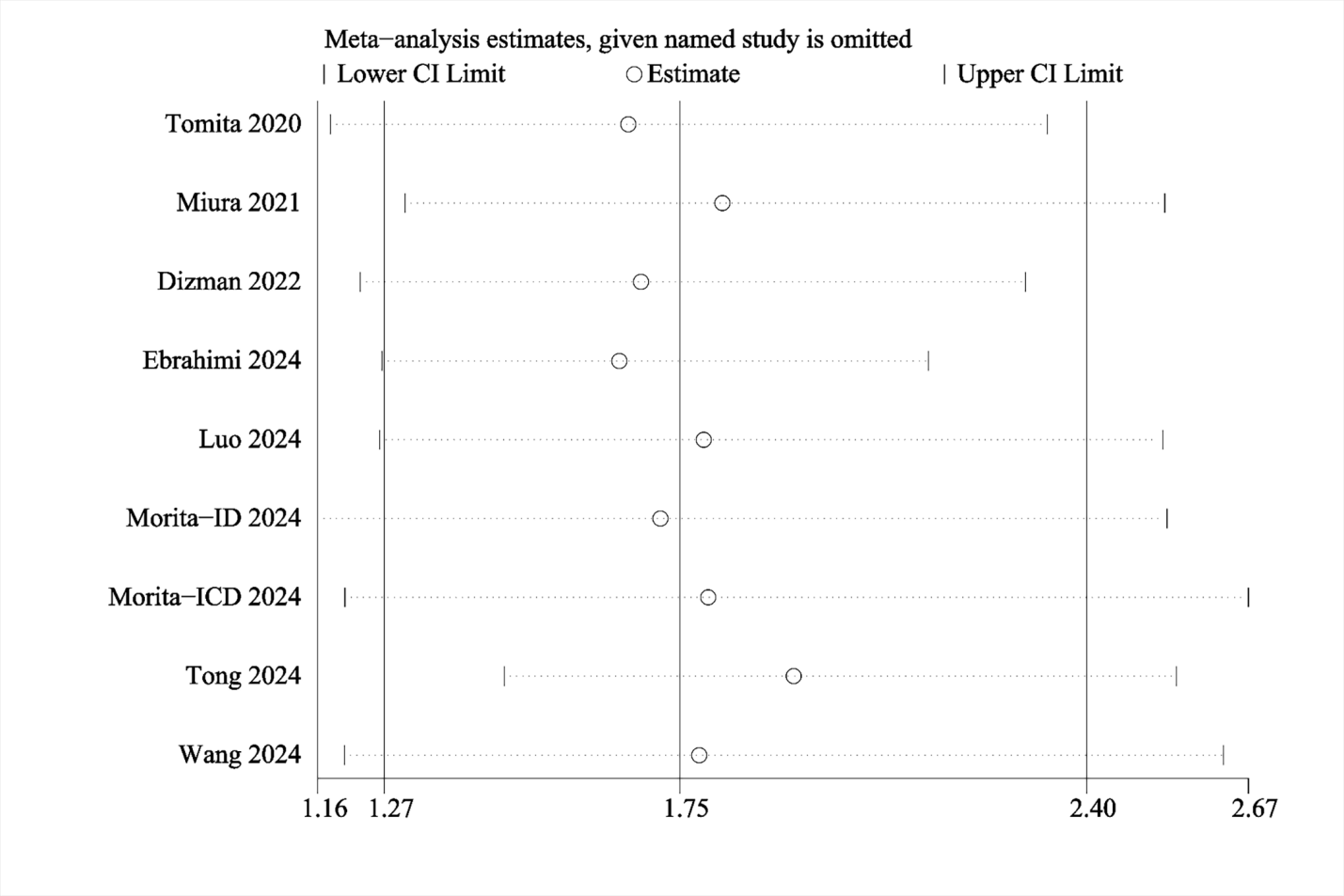


Figure S1H. ORR in NSCLC subgroup


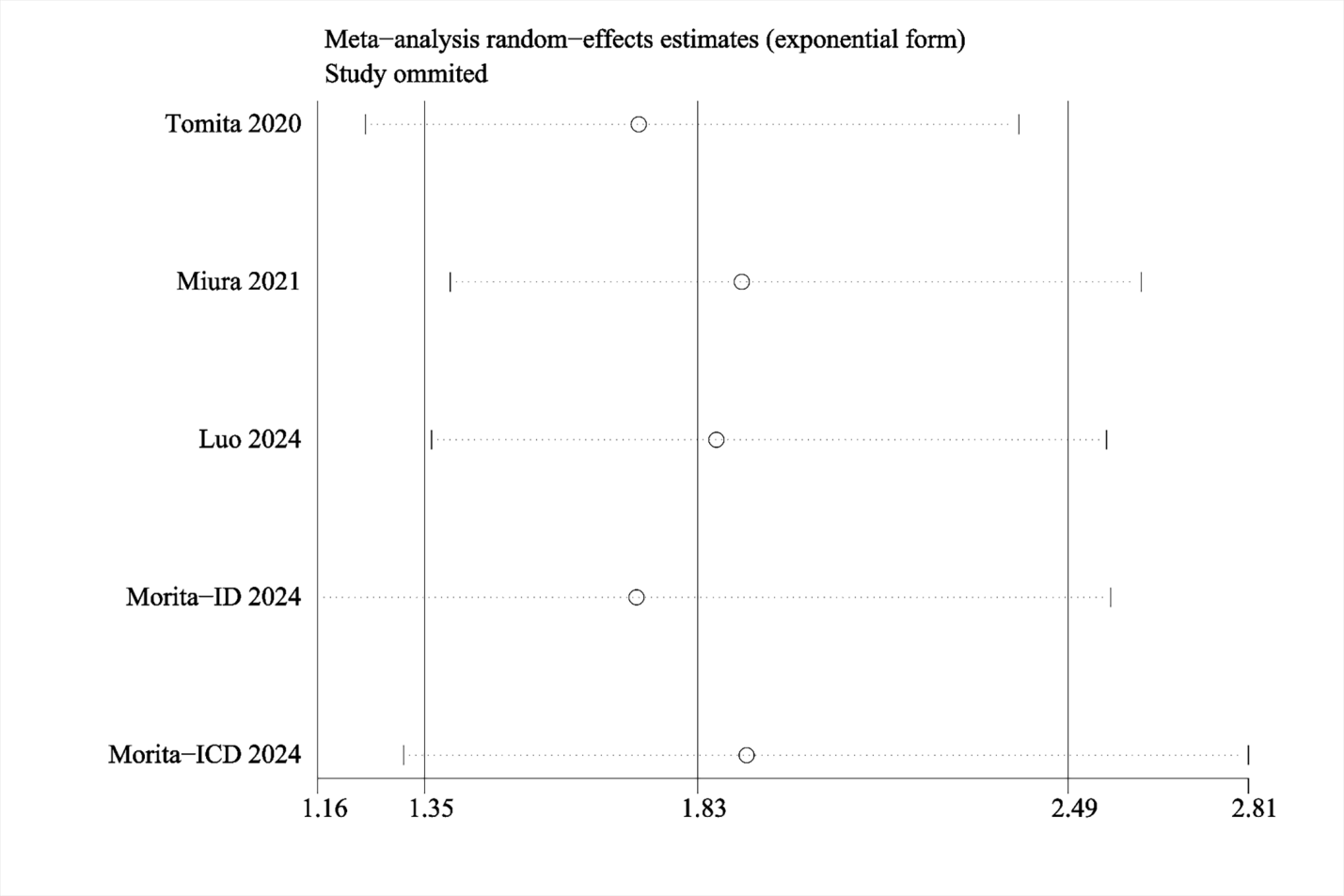


Figure S1I. DCR


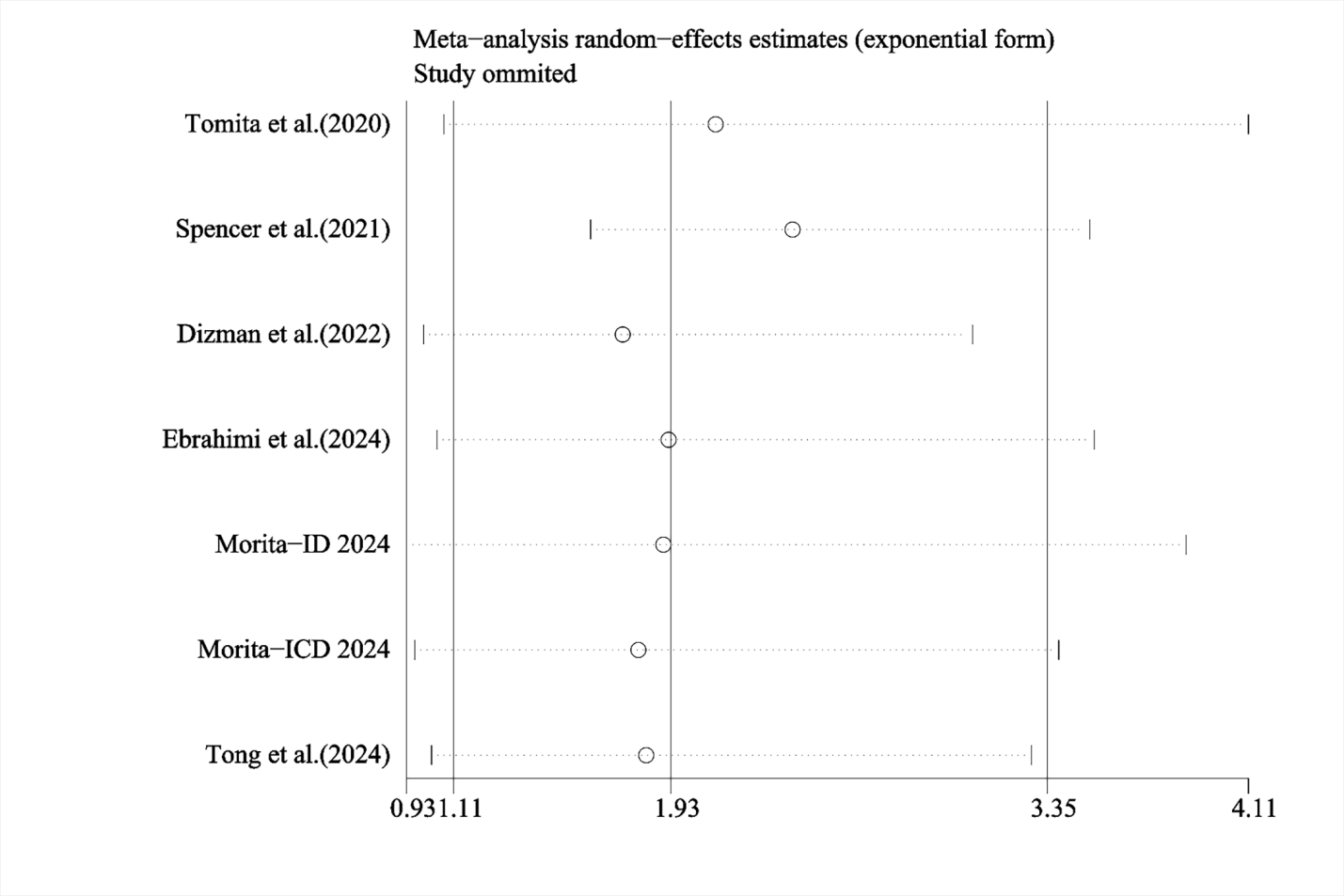


Figure S1J. DCR in NSCLC subgroup


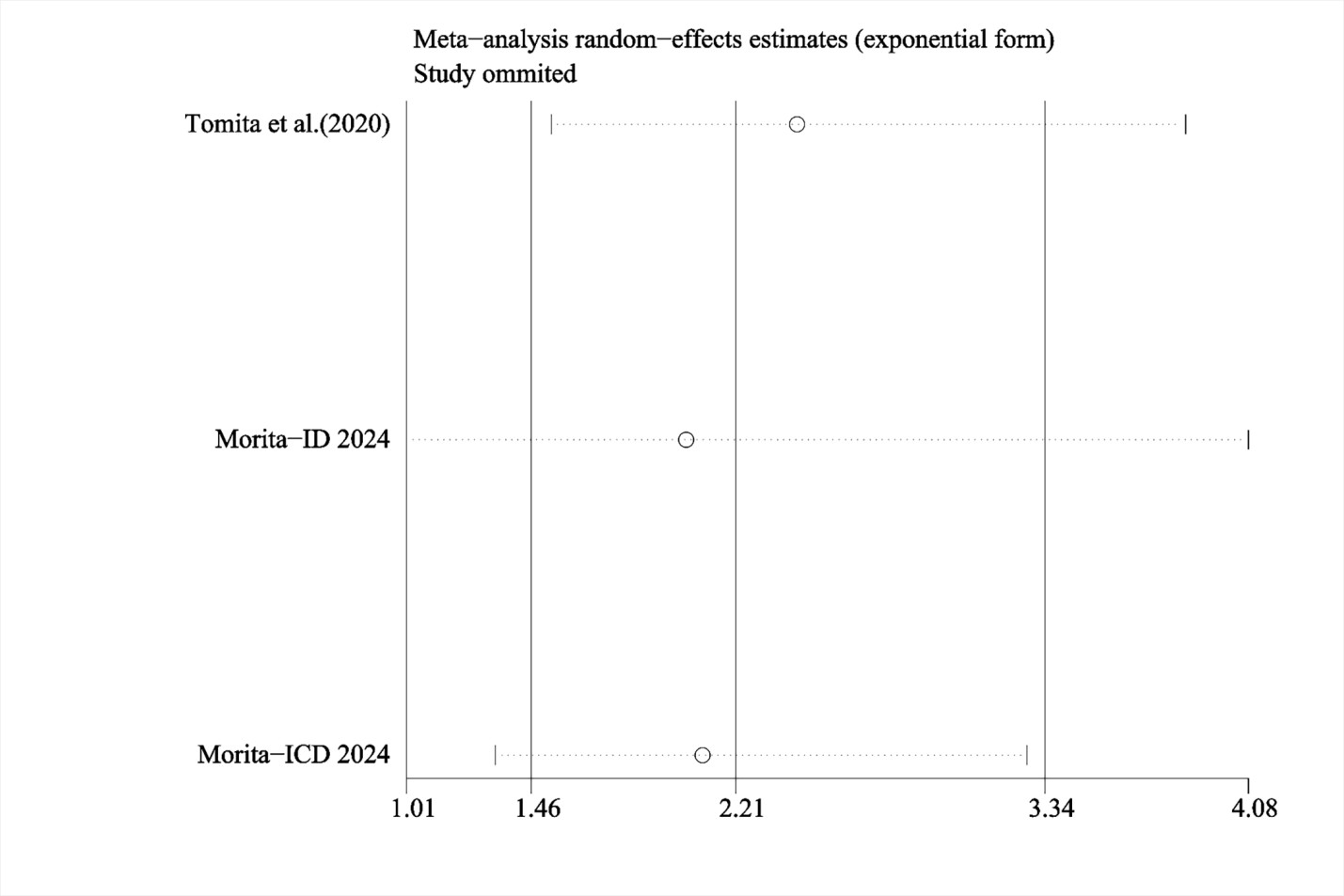


Abbreviations: OS, Overall survival; PFS, progression-free survival, NSCLC, non-small cell lung cancer; OR, odds ratio; ORR, objective response rate, DCR, disease control rate.
